# Subtype-associated epigenomic landscape and 3D genome structure in bladder cancer

**DOI:** 10.1186/s13059-021-02325-y

**Published:** 2021-04-15

**Authors:** Tejaswi Iyyanki, Baozhen Zhang, Qixuan Wang, Ye Hou, Qiushi Jin, Jie Xu, Hongbo Yang, Tingting Liu, Xiaotao Wang, Fan Song, Yu Luan, Hironobu Yamashita, Ruby Chien, Huijue Lyu, Lijun Zhang, Lu Wang, Joshua Warrick, Jay D. Raman, Joshua J. Meeks, David J. DeGraff, Feng Yue

**Affiliations:** 1 Department of Biochemistry and Molecular Biology, Penn State School of Medicine, Hershey, PA USA; 2 Department of Biochemistry and Molecular Genetics, Feinberg School of Medicine Northwestern University, Chicago, IL USA; 3 Present address: Key Laboratory of Carcinogenesis and Translational Research (Ministry of Education/Beijing), Division of Etiology, Peking University Cancer Hospital and Institute, Beijing, China; 4 Department of Pathology and Laboratory Medicine, The Pennsylvania State University, College of Medicine, Hershey, PA USA; 5 Department of Surgery, Division of Urology, The Pennsylvania State University, College of Medicine, Hershey, PA USA; 6 University of Illinois College of Medicine, Chicago, IL USA; 7 Department of Biochemistry and Molecular Biology, The Pennsylvania State University, College of Medicine, Hershey, PA USA; 8 Department of Urology, Feinberg School of Medicine and The Robert H. Lurie Comprehensive Cancer Center, Northwestern University, Chicago, IL USA; 9 Robert H. Lurie Comprehensive Cancer Center of Northwestern University, Chicago, IL USA

## Abstract

**Abstract:**

Muscle-invasive bladder cancers are characterized by their distinct expression of luminal and basal genes, which could be used to predict key clinical features such as disease progression and overall survival. Transcriptionally, FOXA1, GATA3, and PPARG are shown to be essential for luminal subtype-specific gene regulation and subtype switching, while TP63, STAT3, and TFAP2 family members are critical for regulation of basal subtype-specific genes. Despite these advances, the underlying epigenetic mechanisms and 3D chromatin architecture responsible for subtype-specific regulation in bladder cancer remain unknown.

**Result:**

We determine the genome-wide transcriptome, enhancer landscape, and transcription factor binding profiles of FOXA1 and GATA3 in luminal and basal subtypes of bladder cancer. Furthermore, we report the first-ever mapping of genome-wide chromatin interactions by Hi-C in both bladder cancer cell lines and primary patient tumors. We show that subtype-specific transcription is accompanied by specific open chromatin and epigenomic marks, at least partially driven by distinct transcription factor binding at distal enhancers of luminal and basal bladder cancers. Finally, we identify a novel clinically relevant transcription factor, Neuronal PAS Domain Protein 2 (NPAS2), in luminal bladder cancers that regulates other subtype-specific genes and influences cancer cell proliferation and migration.

**Conclusion:**

In summary, our work identifies unique epigenomic signatures and 3D genome structures in luminal and basal urinary bladder cancers and suggests a novel link between the circadian transcription factor NPAS2 and a clinical bladder cancer subtype.

**Supplementary Information:**

The online version contains supplementary material available at 10.1186/s13059-021-02325-y.

## Introduction

Urinary bladder cancers (BLCA) are the second most commonly diagnosed urologic malignancy in the USA, with over 81,400 total new cases diagnosed in 2019 [1, 2]. As BLCA is a morbid disease that is costly to treat, increased molecular understanding is required [3]. Expression of luminal (*FOXA1*, *GATA3*, *PPARG*, etc.) and basal (*KRT1*, *KRT5*, *KRT6A*, etc.) [4, 5] genes have been used to molecularly characterize muscle invasive BLCA. In particular, the presence of basal BLCA, which is often enriched for squamous differentiation, is associated with significant morbidity, disease progression, and lower survival [6, 7].

Recent studies have identified both luminal (FOXA1, GATA3, and PPARG [8]) and basal (TP63 [9–12], STAT3 [4, 13–15], TFAP2A, and TFAP2C [16]) -specific transcription factors (TFs) with functional roles in BLCA. For instance, we previously reported that FOXA1 and GATA3 cooperate with PPARG activation to drive trans-differentiation of basal BLCA cells to luminal subtype [8]. This observation is in agreement with the role of these factors in maintaining urothelial cell differentiation [17, 18] and supports a role for inactivation of FOXA1, GATA3, and/or PPARG during BLCA progression to a basal subtype. Subtype-specific TFs also appear to drive programs in oncogenesis and tumor progression. For example, basal factors STAT3, TP63, and TFAP2A/C have been shown to promote tumor cell invasion and/or metastasis [12, 13, 16]. Similarly loss of FOXA1 [19] and GATA3 [20] have been shown to promote cell migration and invasion. Therefore, specific TFs play a key role in subtype specification.

In addition to directly regulating transcription, studies show that TFs regulate gene expression through epigenetic histone modifications and open chromatin accessibility in breast cancers [21–24]. However, the degree to which the specific repertoire of TFs, epigenetic open chromatin TF accessibility, histone modifications, and 3D genome architecture cooperate for subtype expression is unknown. Therefore, we performed the most comprehensive set of genome-wide experiments to systematically map the epigenome, transcriptome, TF binding, and 3D chromatin loops. To our knowledge, this is the first report identifying 3D genome architecture in bladder cancer. Our work highlights the relevance of epigenetic modifications, open chromatin accessibility, and TF repertoire and identifies a novel identified basic helix loop helix (bHLH) TF NPAS2, all of which cooperate in the coordination of subtype-specific gene expression in bladder cancer.

## Results

### Comprehensive epigenomic profiling in both BLCA lines and primary tumors

**Fig. 1:**
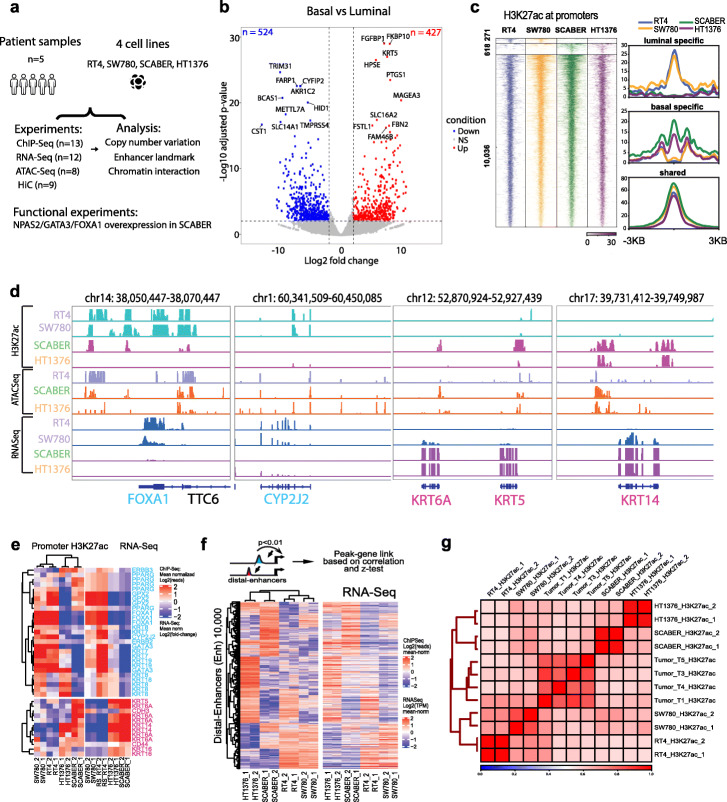
Luminal and basal transcriptional BLCA subtypes are associated with distinct promoter and distal enhancers’ activity at the epigenetic level. **a** Overall design of the study. **b** Differential expression gene (DEG) analysis of luminal cell lines (RT4 and SW780) and basal cell lines (SCABER and HT1376) shows 427 basal-specific upregulated genes and 524 luminal-specific upregulated genes. **c** Heatmap of differential H3K27ac ChIP-Seq at promoters (left). Signal H3K27ac intensity profiles for each cluster of BLCA cells (right). **d** Genome browser signal tracks for a panel of luminal and basal genes. Shown here are the tracks of H3K27ac ChIP-Seq, ATAC-Seq, and RNA-Seq data in RT4, SW780, SCABER, and HT1376 cells. **e** Promoter H3K27ac and its associated RNA-Seq signals for selected luminal and basal genes shows remarkable similarity. **f** Integrated H3K27ac peaks at distal enhancers and RNA-Seq gene expression association model identifies putative enhancers and gene regulation. Top 10,000 most variable enhancers (left heatmap) are plotted along with their corresponding gene expression (right heatmap). **g** Correlations of genome-wide H3K27ac signals between the bladder cancer cell lines and tumor samples demonstrate similarity of enhancer landscape

In this project, we performed RNA-Seq, ChIP-Seq for Histone 3 lysine 27 acetylation (H3K27ac), Assay for Transposase-Accessible Chromatin using sequencing (ATAC-Seq), and genome-wide chromatin confirmation capture experiments (Hi-C) on 4 bladder cancer cell lines (Fig. 1a), two of which (RT4 and SW780) were previously annotated as luminal and the two others (SCABER and HT1376) that were characterized as basal [8, 25]. Based on the RNA-Seq data generated in this study, we used a previously reported molecular subtyping approach [26] to confirm assignment to luminal and basal states. Our results confirmed RT4 and SW780 as belonging to the Luminal-papillary subtype, while SCABER and HT1376 belong to the Basal/squamous subtype (Additional file 1: Table S1). Each experiment in bladder cancer cell lines has at least two biological replicates (Additional file 2: Table S2) and we observed a high correlation between the two replicates (Additional file 3: Table S3). More importantly, we performed the same set of experiments on four patient muscle-invasive bladder tumors as well. By using the same molecular subtyping method, we determined their subtypes as the following: T1 is Luminal-papillary, T3 is Stroma-rich, and T4 and T5 are basal/squamous.

### Luminal and basal transcriptional BLCA subtypes are associated with distinct promoter and distal enhancers activity at the epigenetic level

Enrichment of H3K27ac signals has been used to predict both active promoters and distal enhancers [27, 28]. Therefore, we first performed ChIP-Seq for H3K27ac in all four cell types and four patient samples. We observed that biologic replicates following H3K27ac ChIP-seq always clustered together, indicating our results are highly reproducible (Additional file 4: Figure S1A). Further, we found that two luminal subtypes (RT4 and SW780) clustered together, while two basal (SCABER and HT1376) cell lines are grouped together as well (Additional file 4: Figure S1A). These clustering results suggest global epigenomic profiles accurately reflect cell identity. The hierarchical clustering in the cell lines based on H3K27ac signals was also mirrored by global mRNA expression by RNA-Seq data (Additional file 4: Figure S1B). We performed differential gene expression analysis on the two groups of cell types (RT4 and SW780 vs. SCABER and HT1376) and identified 427 basal-specific (Additional file 5: Table S4) and 524 luminal-specific genes (Fig. 1b, Additional file 6: Table S5).

Next, we examined promoter usage based on H3K27ac signals at known genes. We confirmed that promoter H3K27ac intensities are remarkably similar to gene expression (Fig. 1c), and clustering analysis based on promoter H3K27ac intensity was able to distinguish luminal and basal models of BLCA (Additional file 4: Figure S1C). For example, we observed that two luminal subtype BLCA cell lines RT4 and SW780 have similar H3K27ac patterns at luminal genes *FOXA1*, *GATA3*, and *PPARG* (Fig. 1d, e), while the two basal cell lines share similar promoter marks at genes encoding the basal/squamous markers *KRT5/14*. Interestingly, although based on global gene expression, HT1376 is classified as a basal/squamous subtype, it shows a similar promoter H3K27ac pattern at luminal genes (*GATA3*, *KRT7/8/18*, Fig. 1e).

Distal H3K27ac peaks from gene promoter regions have been used as markers for active enhancers [27, 29]. We took the same approach here, and on average, we predicted 59,466 (40,731–78,506) enhancers in each cell line (Additional file 7: Table S6). To link the distal enhancers to their target genes, we performed a correlation-based distal-enhancer peak-gene association as described in [30] and identified the top 10,000 variable distal enhancers that show significant correlation to its linked gene (correlation ≥0.5, *p* < 0.01; a total of 58,509 satisfied our criteria; Fig. 1f and Additional file 8: Table S7). We observed that the enhancers show clear clustering according to different cell types, and their target genes show similar cell-type-specific patterns (Fig. 1f and Additional file 4: Figure S1D). Moreover, to understand the clinical relevance of our findings, we performed H3K27ac ChIP-Seq in four muscle invasive bladder patient samples. Our results show a remarkable correlation of tumor cell lines (Fig. 1g). In summary, we show in these cell lines and in a limited tumor cohort that epigenetic regulation is correlated with molecular subtype assignment.

### Distinct sets of transcription factor motifs are enriched in luminal and basal BLCA-associated *cis* DNA regulatory regions

**Fig. 2:**
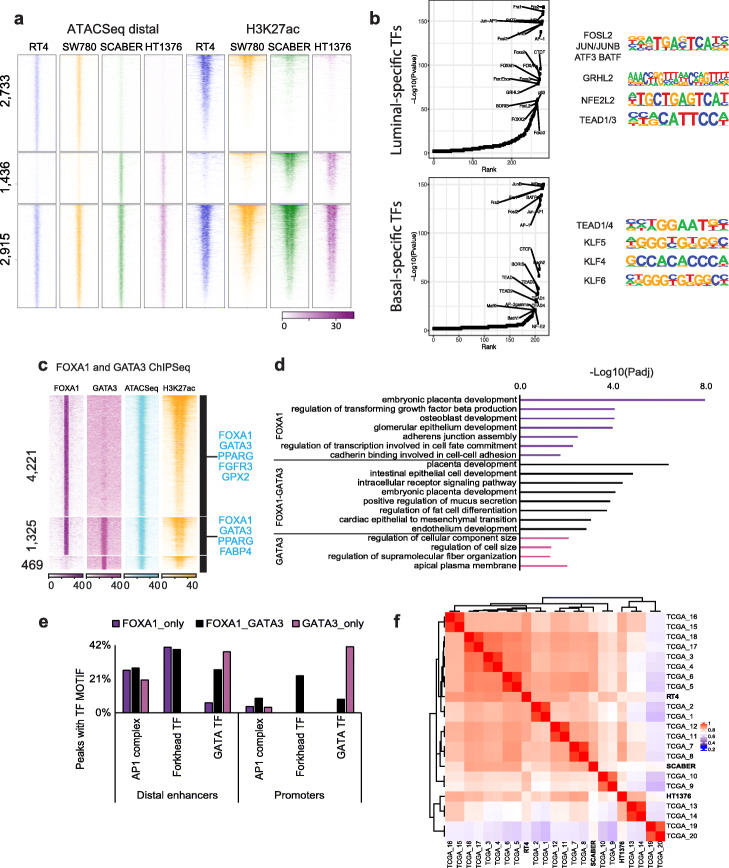
Distinct sets of transcription factor motifs are enriched in luminal and basal BLCA-associated *cis* DNA regulatory regions. **a** A comprehensive and a distinct set of distal ATAC-Seq signals at three clusters (luminal specific, basal specific, and shared) and corresponding H3K27ac signals. **b** TF motif analysis results is shown here as a ranked plot (left) and motifs (right), where for luminal-specific (top) and basal-squamous-specific open chromatin enhancers (bottom). **c** FOXA1 and GATA3 bound open chromatins located at distal enhancers of RT4/luminal cell line is depicted here in three groups: FOXA1 only, GATA3 only, and FOXA1 and GATA3 binding sites. **d** Gene ontology analysis of pathways for each group of binding sites (FOXA1 only, FOXA1 and GATA3, and GATA3 only). **e** Observed occurrence of TF motifs (AP-1, FOX Forkhead, and GATA) is shown here at distal enhancers and promoters of three groups. **f** Genome-wide open chromatins of BLCA cell lines show similarity with TCGA bladder tumors [30]

We performed ATAC-Seq in RT4, SW780, SCABER, and HT1376 cell lines to evaluate their open chromatin status in the genome. On average, in each cell line, we identified 32,000 open chromatin regions (Fig. 2a and Additional file 9: Table S8). Among them, 40.8% of open chromatin regions were located at promoter regions, while 59.2% were located at distal regions. Overall, > 90% of the open chromatin promoter regions overlap with H3K27ac (Additional file 4: Figure S2A, S2C–D). The overlap of distal ATAC-Seq peaks and H3K27ac is lower (Additional file 4: Figure S2A and Additional file 10: Table S9), at least partially due to the different numbers of peaks in different datasets. Genome-wide correlation of ATAC-Seq showed that HT1376 and SCABER clustered together with 80% similarity (Additional file 4: Figure S2E) compared to luminal RT4 (~ 65%). We noted that this observation agrees with the RNA-Seq-based clustering and H3K27ac-based clustering (Additional file 4: Figure S1A and B).

Next, we performed motif analysis of these open chromatin regions (Additional file 11: Table S10). We observed that binding sites for CTCF and AP-1 complex are enriched in all cell lines (Fig. 2b and Additional file 4: Figure S2G). Further ranking of TF motifs by enrichment *p*-value revealed luminal open chromatin regions (shared between RT4 and SW780) were enriched with binding motifs for GRHL2, TP53, and TP63 while basal open chromatins (shared between SCABER and HT1376) were enriched for TEAD1/4 and KLF factor (Fig. 2b) binding motifs. GRHL2 [31] was previously reported to be a luminal gene, thereby validating our findings. Interestingly, binding motifs for AP-1 complex proteins FOSL1/2, JUN/JUNB, ATF3, and BATF TFs [32] were the topmost enriched motifs for both luminal and basal-squamous open chromatins. We then comprehensively mapped all the enriched TF motifs in luminal, basal-squamous and shared open chromatins of distal enhancers to examine the relationship between TFs and BLCA subtypes (Additional file 11: Table S10). We discovered that at distal enhancers, the luminal BLCA subtypes are associated with previously reported steroid hormone receptor TFs. On the other hand, basal-squamous open chromatin areas at enhancers show enrichment of previously unreported factors MADS box TF MEF2C and the homeobox TF OTX2. Not surprisingly, luminal pioneering TFs such as forkhead transcription factors (FOXA1/2/3, FOXF1, FOXK1, FOXM1), and GATA TFs (GATA3/4/6) were enriched in luminal-associated enhancers with an open chromatin conformation. More surprisingly, forkhead and GATA motifs were also identified as being associated with open chromatin at enhancer elements across cell lines (Additional file 11: Table S10). While FOXA1 and GATA3 are known to have low expression in basal bladder cancer cell lines and tumors, the enrichment of forkhead and GATA motifs in open chromatins across BLCA cell lines suggests compensation by Forkhead and GATA factors other than FOXA1 and GATA3. In addition, Forkhead and GATA motif enrichment across cell lines in areas of open chromatin may indicate luminal-specific TFs are poised to bind to these areas of open chromatin. Furthermore, FOXA1 and GATA3 are known to play a role in the development of urothelium [31] suggesting that their binding sites may be primed early during development. We also discovered that the stem-cell-associated pioneering TFs such as KLF factors (KLF10/14), ATF factors (ATF1/2/4/7), and NANOG were enriched in basal-associated enhancers. This is interesting because there exists a progenitor cell population within basal urothelium that can contribute to urothelial development and differentiation [33, 34].

### FOXA1 and GATA3 bind at luminal open chromatins at regulatory distal enhancers to drive expression of luminal-specific genes

We hypothesized that TFs such as FOXA1 and GATA3 bind at the open chromatin region to pioneer luminal enhancers and activate associated gene expression. To test this hypothesis, we performed GATA3 ChIP-Seq in the RT4 luminal BLCA cell line and obtained FOXA1 ChIP-Seq in RT4 cells from our previously published work (Additional file 12: Table S11) [8]. As predicted, luminal TFs FOXA1 and GATA3 showed enriched binding at the open chromatin loci of luminal-associated (*FOXA1*, *GATA3*, *PPARG*, *FGFR3,* and *FABP4*) distal enhancers (Fig. 2c). More specifically, we discovered 1325 distal enhancers that show co-binding of both FOXA1 and GATA3 in RT4 (Fig. 2c). Similarly, FOXA1 and GATA3 showed enriched binding at open chromatin loci of luminal marker genes (*FOXA1*, *ERBB3*, *KRT19*, *GPX2*, and *FABP4*) promoters (Additional file 4: Figure S2F).

GO term analysis of genes proximal to these distal enhancer sites showed regulation of TGF beta production, epithelium development, regulation of transcription involved in cell fate commitment, and cell-cell adhesion biological processes (cadherin binding and adherens junction assembly) as terms associated with FOXA1. In addition, regulation of cellular component, cell size, and apical plasma membrane biological processes were terms identified with GATA3-bound genes proximal to these distal enhancers, suggesting a strong involvement of both TFs in commitment to cell fate and luminal differentiation (Fig. 2d). In regard to proximal genes associated with distal enhancers bound by both FOXA1 and GATA3, terms identified were associated with various developmental processes and the regulation of mucus secretion and fat cell differentiation, both important metabolic attributes of differentiated urothelium (Fig. 2d).

We then proceeded with the motif analysis of FOXA1 only, GATA3 only, and co-bound sites. Surprisingly, AP1-complexes were enriched specifically in all distal enhancers in addition to FOXA or GATA motifs (Fig. 2e). The order of binding of these three factors remains to be investigated. Finally, to understand the clinical relevance of our findings, we compared our four BLCA cell lines to the TCGA muscle-invasive bladder tumor ATAC-Seq data [30] and discovered that the genome-wide open chromatin profile in our cell lines is clustered with distinct clusters of tumors (Fig. 2f), suggesting that the open chromatin regions in these cell lines share similar patterns with patient tumors.

### Luminal and basal subtypes of BLCA show potentially distinct 3D genome organizations

**Fig. 3:**
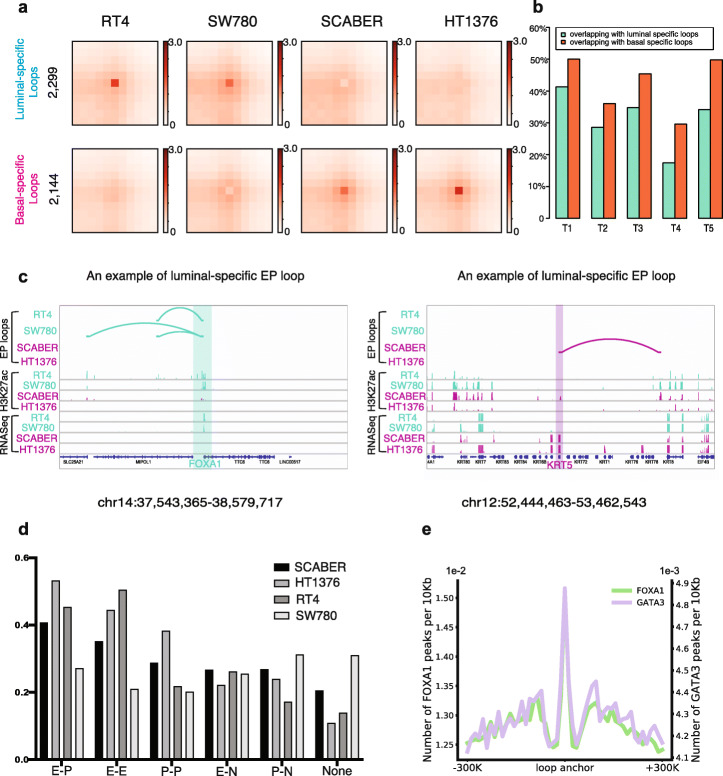
Luminal and basal subtypes of bladder cancers show potentially distinct 3D genome organizations. **a** Hi-C loop analysis of luminal and basal-squamous cell lines show distinct luminal loops and basal-squamous loops. **b** Contacts identified in luminal and basal-squamous cell lines are shared and validated in five bladder cancer tumor samples. **c** Genome-browser tracks for selected luminal gene (FOXA1) and basal gene (KRT5) that contain enhancer-promoter loops are shown here. Arcs indicate the predicted chromatin loops using Hi-C data. **d** The type of contacts based on the overlap of contact location at either enhancer (H3K27ac at distal region) or promoter (H3K27ac and H3K4me3 at promoter) in each cell line is shown. E-P, enhancer-promoter loops; E-E, enhancer-enhancer loops; P-P, promoter-promoter loops; E-N, enhancer-non regulatory loops; P-N, promoter-non regulatory loops; None, non-regulatory loops. **e** Enrichment of FOXA1 (left axis) and GATA3 (right axis) binding sites in RT4 (luminal) cells is shown here at its loop anchors

Previous studies have shown that 3D chromatin organization is associated with epigenetic activation or silencing of genes in cells [35]. For example, the majority of heterochromatin is known to be compressed in nuclei and located near the lamina-associated periphery of the nuclear envelope [35]. To obtain initial insights into the genome-wide 3D landscape of luminal and basal BLCA, we performed high-resolution Hi-C experiments on all four cell lines (at least 800 M reads, each) and five bladder tumor patients (> 800 M reads, each) (Additional file 4: Figure S3). We used our recently developed software, Peakachu [36], which is a machine learning-based chromatin loop detection approach, to predict loops at 10Kb bin resolution. First, we identified an average of 56,315 loops (range between 38,271 and 69,032) in the four cell lines (prob> 0.8; Additional file 13: Table S12). Then, by using the probability score output from Peakachu, we assigned subtype-specific chromatin loops as shown in the Aggregate Peak Analysis (APA, Fig. 3a and Additional file 14: Table S13) [37]. Based on our approach, we observed more potentially luminal-specific loops in RT4 and SW780 (2299) relative to the basal BLCA models SCABER and HT1376 (2144). We then compared each of these categories with loops detected in five patient samples (Fig. 3b): ~ 30–40% of luminal-assigned and basal-assigned 3D chromatin loops identified in the cell lines were observed in these five tumor samples.

Finally, we examined enhancer and promoter loops in each category for their association with subtype-specific gene expression. Examples are shown in Fig. 3c, in which we found that the luminal gene *FOXA1* and the basal gene *KRT5* showed increased number of enhancer-promoter loops in luminal and basal cell lines, respectively. Overall, we observed that ~ 40% of the chromatin loops exist between enhancers and promoters (Fig. 3d). Furthermore, we found a significant enrichment of FOXA1 and GATA3 binding sites at these loop anchors, indicating the involvement of these pioneer factors in the regulation of the 3D genome (Fig. 3e). This finding is in agreement with previous studies reporting the enrichment of FOXA1 binding sites in enhancer-promoter loops [38].

### Copy number variation (CNV) and chromatin loops in bladder cancer

A hallmark of cancer is large structural variations (SVs), which includes inversions, deletions, duplications, and translocations. Recently, it has been shown that alteration in CNVs and SVs can lead to the alterations in 3D genome structure, including the formation of new topologically associated domains (“neo-TADs”) [39] and resultant “enhancer hijacking [40].” Neo-TADs refer to scenarios where an SV event leads to the formation of new chromatin domains, which can in turn affect the expression profiles of the genes located in those regions. In the “enhancer-hijacking” model, altered 3D genome organization results in abnormal enhancer interaction, with enhancers brought in close proximity to the wrong target gene (usually an oncogene) resulting in inappropriate target activation.

**Fig. 4:**
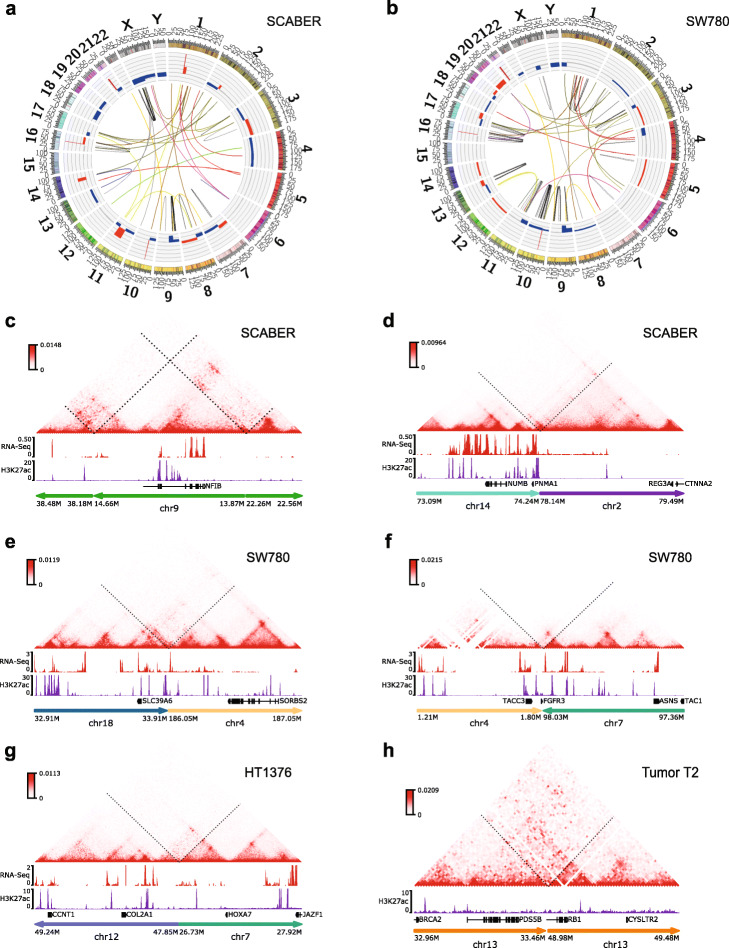
Chromatin interactions induced by structure variation (SV) events. **a**, **b** Circos plot showing intra- and inter-chromosome SVs in SCABER (**a**) and SW780 (**b**). **c** A large intra-chromosomal translocation on chr9. **d**–**h** Inter-chromosomal translocations. The breakpoints were identified by the HiCBreakfinder software. We then reconstructed the local Hi-C maps across the breakpoints. RNA-Seq and H3K27ac ChIP-Seq tracks from the same cell type are shown below the Hi-C maps

We first systematically identified copy number variations (CNVs) and SV events using the Hi-C data with HiNT [41] and the Hi-Cbreakfinder [42] software. We identified tens of large SVs, including inversions, deletions, and translocations (Fig. 4a, b, Additional file 4: Figures S4–S5, Additional file 15: Table 14). As might be expected, we observed fewer CNVs in the patient samples than in cell lines. More importantly, we were able to re-construct the local Hi-C map surrounding the breakpoints of the SVs. We can observe interesting enhancer-hijacking events and the formation of neo-TADs in these local Hi-C maps (Fig. 4c–h). These observations provide an important resource to further study the function of the re-arranged enhancers in the context of bladder cancer.

### Neuronal PAS Domain Protein 2 (NPAS2) is a novel luminal BLCA TF which regulates luminal gene expression and cell migration

**Fig. 5:**
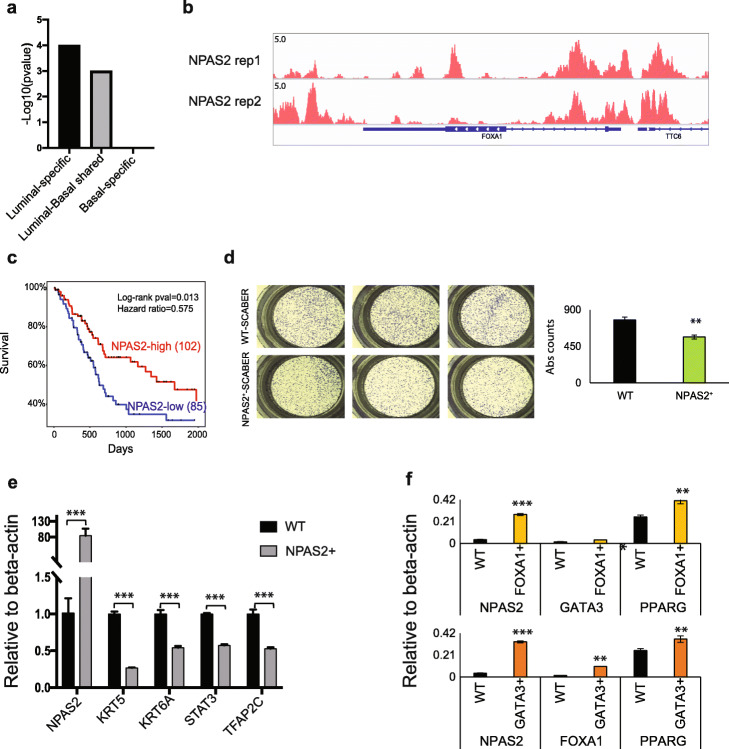
NPAS2 is a novel bladder cancer regulator. **a** *p*-values of NPAS2 motif in luminal-associated (RT4, SW780), basal-associated (SCABER, HT1376), and shared open chromatin regions. **b** NPAS2 ChIP-seq signal near luminal marker genes *FOXA1*, *GATA3*, and *PPARG* in HEPG2 cell line. **c** NPAS2 Kaplan-Meier curve is shown here for 2000 days with log-rank statistics and hazards ratio. **d** Transwell migration assay representative crystal violet staining (left) and quantification of differences in transwell migration (right) are shown following overexpression of NPAS2 in SCABER. **e** RT-qPCR results for basal marker genes *KRT5*, *KRT6A*, *STAT3*, and *TFAP2C* are shown here for wild-type and NPAS2 overexpressed SCABER basal cell line. **f** *NPAS2*, *FOXA1/GATA3*, and *PPARG* RT-qPCR are shown here for wildtype and FOXA1/GATA3 overexpressed SCABER basal cell line

Genome-wide open chromatin analysis of BLCA cell lines provides an ideal platform for the identification of novel transcriptional regulators of BLCA cell fate and phenotype. Here we performed motif analysis of luminal-associated, basal-associated, and shared open chromatin regions, resulting in the identification of distinct TFs in each cluster. Among them, many represent known families of subtype-specific regulators, such as the GATA, FOX, and ETS families at luminal-associated ATAC-Seq peaks. Among them, we noticed a potential novel bHLH containing regulator, NPAS2, which is enriched in the luminal-associated and shared clusters, but not enriched in basal-associated ATAC-Seq peaks (Fig. 5a). We examined its binding profile using the latest ENCODE data (HEPG2 cells) [43] and found that NPAS2 binds at the FOXA1 promoter region (Fig. 5b), but not at regulatory regions for basal marker genes. This suggests the possibility that NPAS2 may be an upstream regulator of FOXA1. We then checked the TCGA data and found that high expression level of NPAS2 is significantly correlated to overall patient survival (Fig. 5c).

To further determine whether NPAS2 expression influences the downstream target expression and phenotype, we overexpressed NPAS2 in the basal-squamous BLCA cell line SCABER. First, we performed trans-well migration assays and found that overexpression of NPAS2 in SCABER cells decreased cell trans-well migration (Fig. 5d). We then performed RT-qPCR experiments and found that the basal marker genes (such as *KRT5*, *KRT6A*, and *TFAP2C*) are significantly downregulated (Fig. 5e) following NPAS2 overexpression, suggesting NPAS2 represses the expression of a subset of basal marker genes.

Because our functional genomics analysis suggests that FOXA1 and GATA3 cooperate to regulate luminal target genes [8], we individually overexpressed FOXA1 and GATA3 in SCABER cells to test their ability to regulate NPAS2 expression. We observed increased expression of NPAS2 by both FOXA1 and GATA3 overexpression (Fig. 5f).

## Discussion

Muscle invasive BLCA is a morbid and expensive disease to treat [3, 44–46]. However, with recent development of immunotherapies such as anti-PD-1 [47] and PD-L1 [48], as well as targeted approaches including FGFR3 inhibitors, clinical management has been revolutionized [49]. However, response rates to these and other standard approaches are suboptimal, suggesting the need for increased molecular understanding. In keeping with this, recent National Comprehensive Cancer Network (NCCN) guidelines have encouraged biomarker and molecular-based subtype studies to further stratify patients for recent targeted therapies [50].

It has been suggested that RNA-Seq-based molecular subtyping of BLCA is prognostic of clinical outcomes in patients [6–8]. While TCGA and other studies have identified mRNA-based molecular subtypes, the epigenetic differences underlying these expression subtypes are unknown. The Encyclopedia of DNA Elements (ENCODE) Consortium has contributed greatly to the current understanding of how epigenetic modifications in multiple tissues vary to regulate tissue-specific gene expression [29]. Histone modification states such as H3K27ac, among various other epigenetic states, mark enhancers and promoters that form a complex interacting network hub to regulate gene expression [51, 52]. TCGA has incorporated DNA methylation to understanding epigenetic states [53]. DNA methylation states have been shown to be coupled with histone modifications particularly at the CpG cites at promoters [54]. However large changes in epigenetic histone modification states that influence gene expression lie in distal regions in enhancers and other sites that orchestrate the 3D genome (CTCF) [29, 38, 55–57]. Hence, our study utilized large-scale genomic experiments such as ATAC-Seq and H3K27ac ChIP-Seq, as well as FOXA1 and GATA3 ChIP-Seq and Hi-C combined with RNA-Seq to construct a comprehensive molecular map of luminal and basal BLCA in both cell line models as well as patient tumors. We have further utilized TCGA datasets to orthogonally validate and derive inferences for clinical importance of our findings.

We found evidence for regulation of luminal and basal bladder cancer genes by proximal-promoters and distal enhancers that form long-range chromatin loops and potentially drive oncogenic programs. Our findings are largely in agreement with previously known work on the role of FOXA1 and GATA3 in the regulation and maintenance of oncogenic programs in luminal bladder cancers [8, 19–21]. Interestingly, we found a novel co-regulation of FOXA1 and GATA3 with a common binding partner of AP-1 complex like breast cancers [58, 59] that appears to drive activity of distal enhancers, but not promoters. Our comprehensive 3D genome map shows distinct chromatin loop interaction networks available to luminal and basal BLCA. To our knowledge, this is the first report of a comprehensive 3D genomic map of bladder tumor patients. Our analysis further demonstrates the regulation of distal enhancers with promoters of BLCA subtype-specific genes through physical loops as reported in other studies [37, 38, 52]. Through our analysis, we have identified a novel bHLH TF regulator of luminal BLCA—NPAS2—whose expression correlates with overall survival of BLCA patients included in the TCGA cohort. Through several biological experiments, we showed that NPAS2 regulates the expression of several genes which serve as markers of basal-squamous BLCA, and further diminishes the migration ability of basal BLCA cells. Most importantly, our work highlights how these TFs can cooperatively regulate molecular subtypes and drive clinical associations.

The clinical implication of identification of potential regulators of primary luminal differentiation such as NPAS2 is that, once identified, these factors can be leveraged or targeted. In cases in which the cancers are less luminal and more basal, shifting the biology of the tumor to a more luminal gene expression subtype by activating NPAS2 (and other required factors) could slow the growth of a basal tumors. Alternatively, 30% of muscle invasive tumors are luminal, with upregulated luminal pathways, and blocking the function of luminal tumors could potentially improve survival in patients with luminal BLCA.

Although our studies provide an excellent starting point by identifying associations between epigenetic landscape and 3D genome architecture with tumor subtype, increased numbers of tumor specimens will be required. However, the cost of sequencing limited our current capability to include a large set of patient tumors. An additional limitation of our current study is the lack of a consortium-level analysis of ChIP-Seq data for histone modifications and all major TFs. Such an approach would increase precision in our regulatory analysis. However, previous studies were limited to the understanding of single or combination of few TFs in the context of gene regulation. Therefore, we believe that our study will emerge to be a solid comprehensive resource to launch a further series of hypothesis-driven biological experiments based on gene and epigenetic perturbations to unveil both novel molecular targets as well as biomarkers.

## Materials and methods

### Cell lines and patient tumor samples

Bladder cancer cell lines—RT4, SCABER, SW780, and HT1376—were obtained from ATCC and cultured as previously described [8]. Bladder tumor samples were obtained from Penn State Hershey, College of Medicine’s biobank storage at the Institute of Personalized Medicine (IPM) with appropriate protocol approval from the institutional review board (IRB Number: STUDY00001117). The samples from Northwestern University were also obtained with proper approval from the institutional board (IRB number: STU00088853). Samples were selected based on its availability (50 mg) for several rounds of sequencing experiments.

### Cell culture

Bladder cancer cell lines were cultured in growth medium containing media + 10% fetal bovine serum and 1% penicillin and streptomycin (Corning). RT4, SW780, SCABER, and HT1376 cells were cultured in McCoy’s 5A (Gibco), RPMI-1640 (Corning), Eagle minimum essential medium (MEM; GE life sciences), and Eagle MEM with 1% non-essential amino acids (Corning), respectively. Cells were plated in tissue culture plates (TCPs, Corning)—T-25, T-75 or 15-cm dishes to further grow and expand in 5% CO_2_ humidified incubator for several different sequencing experiments. For future storage, cells were preserved in 5% DMSO containing growth medium in the vapor of liquid nitrogen. For passaging, cells were washed with phosphate-buffered saline (PBS, Corning) and trypsinized for 5 min to detach cells from the TCPs. They were further spun down at 200×*g* to pellet and washed with PBS for further experiments.

### RNA-Seq

For RNA-Seq, RNA was extracted from frozen cell pellets using RNeasy Mini Kit (Qiagen). Extracted RNA was quantitated using NanoDrop (Thermo Scientific). SureSelect strand-specific RNA library preparation kit (Agilent) was used to generate cDNA libraries where polyA RNA was pulled down using 2 μg of oligo (dT) beads. Extracted cDNA was then fragmentation, reverse transcribed, end repaired, 3′-end adenylated, adaptor ligation, and subsequently amplified and beads purified (Beckman Coulter). Barcode sequences were thus used to multiplex high-throughput sequencing. The cDNA library was QC’ed for size distribution and concentration using BioAnalyzer (Agilent) High Sensitivity DNA Kit (Agilent) and Kapa Library Quantification Kit (Kapa Biosystems). Final libraries were then pooled and diluted to 2 nM and subsequently sequenced using Illumina HiSeq, X Ten, or NovaSeq platform (Illumina).

### Chromatin crosslinking and ChIP-Seq library preparation

Each cell line was grown in 15-cm dishes (× 4) with 25-mL growth medium where they were detached from the TCPs using trypsin as described above and further pelleted. Approximately 10–20 M cell pellets (2x biological replicates) were crosslinked immediately with 1% formaldehyde in PBS at RT for 10 min and subsequently quenched with 0.125 M glycine for 5 min. Crosslinked cells were then washed in PBS and 100 μL freshly prepared lysis buffer (1% SDS, 50 mM Tris-HCl pH 8, 20 mM EDTA, and 1x complete protease inhibitor) was added. Lysed cells were then diluted in 900 μL TE buffer and sonicated using focused beam ultrasound sonicator (COVARIS). Sonicated samples were repeated for extended periods of time (up to 1.5 h) until the chromatin size distribution of ~ 200–300 bp was achieved. Sonicated DNA-chromatin complexes were then pulled down with anti-H3K27ac antibody and washed several times with RIPA buffer to remove non-specific bindings. Pulled-down samples as well as input controls were all de-crosslinked at 65 °C overnight. Samples were treated with RNase and Proteinase K digestion at 37 °C and 55 °C, respectively, followed by further DNA library extraction using phenol-chloroform method. The library was then prepared using Kapa Hyper Prep Kit (KAPA) and further amplified using Hi-fidelity KAPA PCR kit (KAPA) for 6–11 cycles and purified with Kapa pure beads (KAPA). The final library was quantified using Qubit high sensitivity DNA assay (Thermofisher) and then sequenced using Illumina’s HiSeq platform 2500, X Ten, or NovaSeq platform (Illumina).

### Nuclei extraction and ATAC-Seq library preparation

Each cell line was pelleted following detachment from the TCPs, as described above. Cells were washed with PBS, counted and kept on ice as a pellet. Fifty thousand cells were used for ATAC-Seq library preparation as described by Greenleaf [60]. First, pelleted cells were reconstituted in a freshly made lysis buffer to remove unwashed mitochondria DNA, spun down, and the buffer was discarded. Then, the pelleted nuclei were tagmented with Tn5 transposase (Illumina ref: 15027866 & 15027865) in 50 μl volume for 30 min at 37 °C and the DNA was subsequently purified using Qiagen MinElute kit (Qiagen). Then, the library was amplified using Hi-fidelity KAPA PCR kit (KAPA) with Nextera’s PCR nonbarcoded Ad1 and barcoded Ad2.* primers for 6 cycles and purified using Kapa pure beads (KAPA). The library was then quantified using Qubit high sensitivity DNA assay (Thermofisher) and further assessed for quality using bioanalyzer (Agilent). Libraries were either sequenced using Illumina’s HiSeq platform 2500, X Ten, or NovaSeq platform (Illumina).

### Chromatin crosslinking and HiC library preparation

Each cell line was grown in T-25 flasks (× 4) with 5-mL growth medium, trypsinized as described above, and pelleted. Approximately 4–5 M cells as pellets were crosslinked immediately with 2% formaldehyde in PBS at RT for 10 min. Crosslinked cells were then washed in PBS and frozen as 1–1.5 M aliquots in −80 °C for several months before library preparation. We used ARIMA’s Hi-C kit for making libraries (ARIMA Genomics). As per their protocol, we tested and did QC on samples and sequenced 300 M–600 M reads for each sample using Illumina’s NovaSeq platform (Illumina).

### Overexpression of NPAS2

Overexpression of FLAG (DYKKK) tagged NPAS2 protein was performed using genscript plasmids, which were expanded following transformation in in competent *Escherichia coli* and picking clones. We then transiently transfected SCABER cell lines with 2 μg plasmid/well in 6-well plates (2x wells). For transfection, we used Lipofectamine 3000 reagent (Invitrogen) as per manufacturer protocol and allowed cells to be cultured up to 2 days before collecting it for various analysis.

### Quantitative reverse transcription PCR (RT-qPCR)

We used the following primers for RT-qPCR to detect mRNA levels.

NPAS2: 5′-ACACCCTTTCAAGACCTTGCC-3′ (F); 5′-AGGTTCGTCAACTATGCACATTT-3′ (R)

FOXA1: 5′-CGCTTCGCACAGGGCTGGAT-3′ (F); 5′-TGCTGACCGGGACGGAGGAG-3′ (R)

GATA3: 5′-ATACACCACCTACCCGCCTAC-3′ (F); 5′-ACTCCCTGCCTTCTGTGCT-3′ (R)

PPARG: 5′-GATCTCCAGTGATATCGACCAGC-3′ (F); 5′-GATGGCCACCTCTTTGCTCTG-3′ (R)

TFAP2C: 5′- ATCGAAAAATGGAGGCCGGT-3′ (F); 5′-CGGCTTCACAGACATAGGCA-3′ (R)

STAT3: 5′- GAGGACTGAGCATCGAGCA-3′ (F); 5′-CATGTGATCTGACACCCTGAA-3′ (R)

KRT5: 5′- GATCGCCACTTACCGCAAGC-3′ (F); 5′-ACTGCCATATCCAGAGGAAACA-3′ (R)

KRT6A: 5′-AAGTGTTGTGAACCCCCACCC-3′ (F); 5′-AGCAATTGCAAACAGCGAAGAG-3′ (R)

beta-actin: 5′-CATGTACGTTGCTATCCAGGC-3′ (F); 5′-CTCCTTAATGTCACGCACGAT-3′ (R)

RNA was extracted from frozen cell pellets using RNeasy Mini Kit (Qiagen). Samples were treated with DNase to digest any additional DNA extracted during the process. DNase-free RNA was then further converted to cDNA using reverse transcriptase kit (Invitrogen) according to the manufacturer’s protocol. Reverse transcribed cDNA was then assayed for RT-qPCR using KAPA SYBR FAST qPCR Master Mixes (Roche) at 60 °C melting temperature and quantitated using BioRad quantitative PCR system. CT values obtained through the quantitation were then normalized to beta-actin and further transformed to relative expression shown in plots.

### Transwell migration assay

Transwell migration assay was performed using 8 μm PVDF inserts (Corning) in a transwell chamber fitting into 24-well plates (Corning). Each cells (control and overexpressing NPAS2 for 2 days) were seeded with 50,000 cells in a transwell (× 3 replicates) in a FBS-free medium containing 1%PS. Cells were allowed to migrate through the transwell inserts for 24 h into medium containing regular 10% FBS and 1%PS. Transwell chambers were removed, washed with PBS and stained with crystal violet. Cells that were not migrated through the insert were removed using a Q-tip. Migrated cells were then visualized in microscope and scored for number of stained spots and compared with different experiment groups. *T*-test was used to calculate significance between groups.

*Computational analysis methods* are provided in Supplementary information part as Additional file 16.

## Supplementary Information

**Additional file 1: Table S1.** Classification results for each sample using consensusMIBC.

**Additional file 2: Table S2.** Cell Lines and Experiment table.

**Additional file 3: Table S3.** RNA-Seq correlation between cell lines.

**Additional file 4: Figure S1.** Epigenetic landscape analysis of histone modifications in luminal and basal bladder cancers. **a** Genome-wide H3K27ac signals show that biological replicates and molecular subtypes (basal and luminal) cluster together. **b** Hierarchical clustering of genome-wide RNA-Seq results for 4 cell lines recapitulate the luminal and basal gene expression based molecular subtypes. **c** Integrated H3K27ac peaks at promoters and RNA-Seq gene expression association model identifies putative promoter and gene regulation. Top 10,000 most variable promoters (left heatmap) are plotted along with their corresponding gene expression (right heatmap). Luminal (cyan) and basal (magenta) genes are highlighted for their specific linked enhancers. **d** Corresponding enhancer H3K27ac and its linked RNA-Seq signals based on our predicted model for selected luminal and basal genes shows remarkable similarity. **Figure S2.** Epigenetic landscape analysis of open-chromatins in luminal and basal subtypes of bladder cancers. **a** Genome-wide overlap of ATAC-Seq peaks with H3K27ac ChIP-Seq is shown here for each cell line at either promoter, enhancer or all locations. **b** Genome-wide overlap of H3K27ac ChIP-Seq peaks with ATAC-Seq is shown here for each cell line at either promoter, enhancer or all locations. **c** Overlap between distal H3K27ac and ATAC-Seq peaks. **d** ATAC-Seq signal at distal enhancers compared with distal H3K27ac signal. **e** Genome-wide correlation of ATAC-Seq signals between cell lines recapitulate enhancer/promoter and RNA-Seq based clustering. **f** FOXA1 and GATA3 ChIP-Seq binding sites overlapped at promoters are shown here as genome-wide tag plot in three groups. **g** A comparison of top 3 motifs enriched *p*-values in each open-chromatins that does not overlap with any H3K27ac signals within its cell lines are shown. **Figure S3.** Hi-C maps of luminal and basal subtypes of bladder cancers and bladder tumors. Genome-wide chromosome view of Hi-C map is shown for RT4 (**a**), SW780 (**b**), SCABER (**c**), HT1376 (**d**), tumor T1 (**e**), tumor T2 (**f**), tumor T3 (G), tumor T4 (H) and tumor T5 (I) at 10 MB resolution. **Figure S4.** Copy number profiles for four bladder cancer cell lines (HT1376, RT4, SW780, and SCABER) and five tumor samples (Tumor T1, Tumor T2, Tumor T3, Tumor T4, and Tumor T5). CNVs were computed using Hi-C data. **Figure S5.** Intra- and inter-chromosome structure variation (SV) events. Circos plot showing intra- and inter-chromosome SVs in HT1376 (**a**), RT4 (**b**), Tumor T1 (**c**), Tumor T2 (**d**), Tumor T3 (**e**), Tumor T4 (**f**) and Tumor T5 (**g**).

**Additional file 5: Table S4.** Basal up-regulated genes from differentially expressed genes (DESeq2) analysis.

**Additional file 6: Table S5.** Luminal up-regulated genes from differentially expressed genes (DESeq2) analysis.

**Additional file 7: Table S6.** H3K27ac ChIP-Seq peaks called in each cell line and its proximal/distal location w.r.t transcription start site (TSS).

**Additional file 8: Table S7.** Epigenetic landscape of H3K27ac signals correlated and linked to nearby genes.

**Additional file 9: Table S8.** ATAC-Seq peaks called in each cell line and its proximal/distal location w.r.t transcription start site (TSS).

**Additional file 10: Table S9.** ATAC-Seq clusters representing Luminal-specific, Basal-specific and shared open-chromatins at distal-enhancers.

**Additional file 11: Table S10.** TF motif analysis of luminal specific, basal specific and shared open-chromatin. Clusters at distal-enhancers.

**Additional file 12: Table S11.** FOXA1 and GATA3 ChIP-Seq peaks called in RT4 cells.

**Additional file 13: Table S12.** Peakachu Loops called in each bladder cancer cell line.

**Additional file 14: Table S13.** Luminal-specific and Basal-specific loops.

**Additional file 15: Table S14.** Number of large structural variations (SVs) in bladder cancer cell lines and tumor samples.

**Additional file 16.** Computational data analysis methods.

**Additional file 17.** Review history.

## Data Availability

Our data is available in gene expression omnibus (GEO) at GSE148079 [61]. Link to data: https://www.ncbi.nlm.nih.gov/geo/query/acc.cgi?acc=GSE148079. Raw fastQ files for patient samples are available in European Genome-phenome Archive (EGA) at EGAS00001005071 [62]. Link to data: https://ega-archive.org/studies/EGAS00001005071. The TCGA ATAC-seq datasets in Fig. 2f are from [30]. The TCGA datasets we used in Fig. 5c are from [53]. The analysis code for figures could be found in GitHub repository (https://github.com/Qixuan771/Source-code-for-bladder-cancer-project, under GNU General Public License v3.0) [63] and zenodo https://zenodo.org/record/4396080#.X-oRtOlKgY0, DOI: 10.5281/zenodo.4396080) [64].
