# Supplementary material for: Subtype-associated epigenomic landscape and 3D genome structure in bladder cancer
Source: Genome Biol. 2021 Apr 15;22:105. doi: 10.1186/s13059-021-02325-y (PMC8048365; doi:10.1186/s13059-021-02325-y)
Supplement: Supplementary file 4 — Additional file 4: Figure S1. Epigenetic landscape analysis of histone modifications in luminal and basal bladder cancers. a Genome-wide H3K27ac signals show that biological replicates and molecular subtypes (basal and luminal) cluster together. b Hierarchical clustering of genome-wide RNA-Seq results for 4 cell lines recapitulate the luminal and basal gene expression based molecular subtypes. c Integrated H3K27ac peaks at promoters and RNA-Seq gene expression association model identifies putative promoter and gene regulation. Top 10,000 most variable promoters (left heatmap) are plotted along with their corresponding gene expression (right heatmap). Luminal (cyan) and basal (magenta) genes are highlighted for their specific linked enhancers. d Corresponding enhancer H3K27ac and its linked RNA-Seq signals based on our predicted model for selected luminal and basal genes shows remarkable similarity. Figure S2. Epigenetic landscape analysis of open-chromatins in luminal and basal subtypes of bladder cancers. a Genome-wide overlap of ATAC-Seq peaks with H3K27ac ChIP-Seq is shown here for each cell line at either promoter, enhancer or all locations. b Genome-wide overlap of H3K27ac ChIP-Seq peaks with ATAC-Seq is shown here for each cell line at either promoter, enhancer or all locations. c Overlap between distal H3K27ac and ATAC-Seq peaks. d ATAC-Seq signal at distal enhancers compared with distal H3K27ac signal. e Genome-wide correlation of ATAC-Seq signals between cell lines recapitulate enhancer/promoter and RNA-Seq based clustering. f FOXA1 and GATA3 ChIP-Seq binding sites overlapped at promoters are shown here as genome-wide tag plot in three groups. g A comparison of top 3 motifs enriched p-values in each open-chromatins that does not overlap with any H3K27ac signals within its cell lines are shown. Figure S3. Hi-C maps of luminal and basal subtypes of bladder cancers and bladder tumors. Genome-wide chromosome view of Hi-C map is shown for RT4 (a), SW780 (b), SCA [file 13059_2021_2325_MOESM4_ESM.pdf]

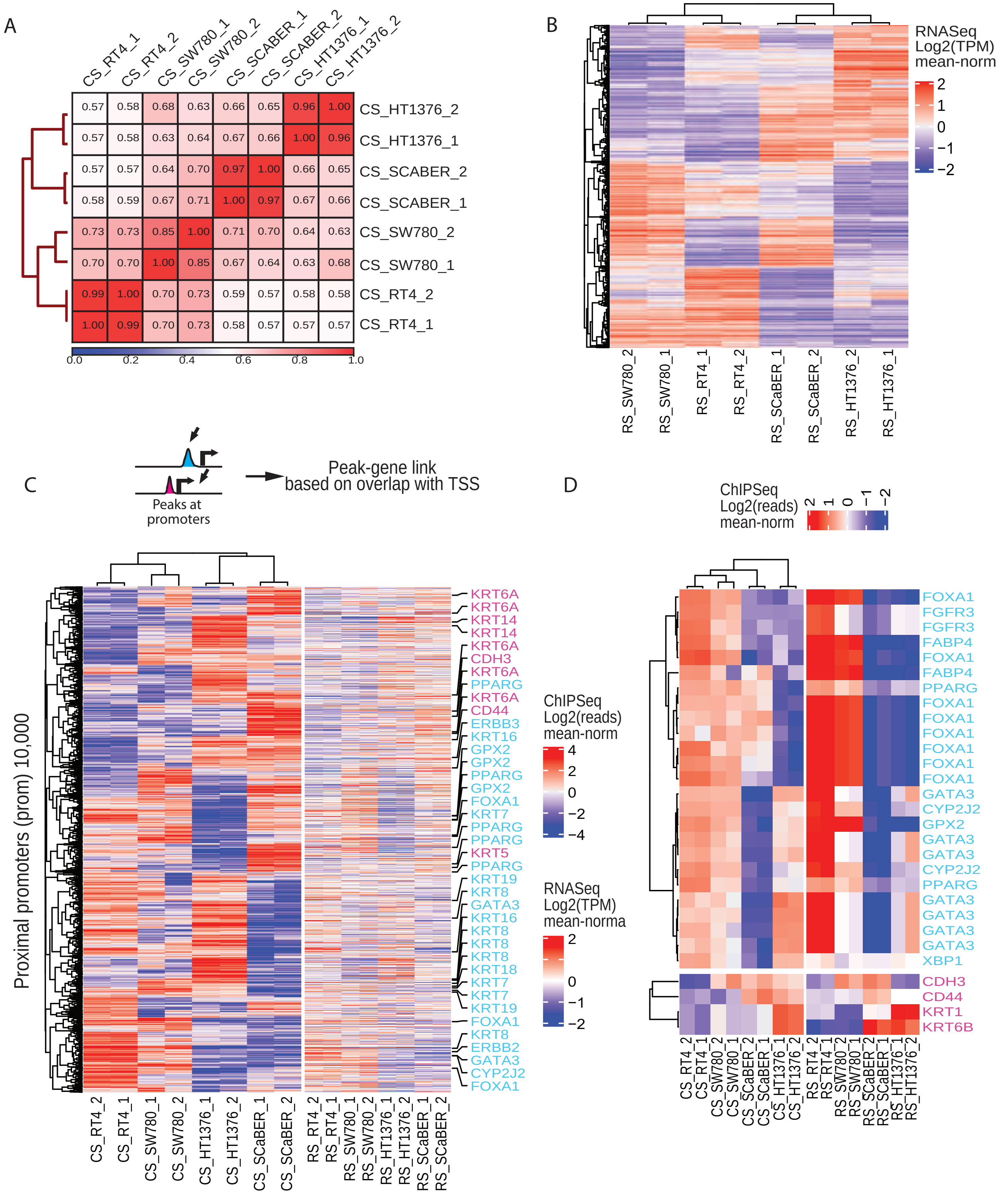

Figure S1: Epigenetic landscape analysis of histone modifications in luminal and basal bladder cancers. A) Genome-wide H3K27ac signals show that biological replicates and molecular subtypes (basal and luminal) cluster together. B) Hierarchical clustering of genome-wide RNA-Seq results for 4 cell lines recapitulate the luminal and basal gene expression based molecular subtypes. C) Integrated H3K27ac peaks at promoters and RNA-Seq gene expression association model identifies putative promoter and gene regulation. Top 10,000 most variable promoters (left heatmap) are plotted along with their corresponding gene expression (right heatmap). Luminal (cyan) and basal (magenta) genes are highlighted for their specific linked enhancers. D) Corresponding enhancer H3K27ac and its linked RNA-Seq signals based on our predicted model for selected luminal and basal genes shows remarkable similarity.

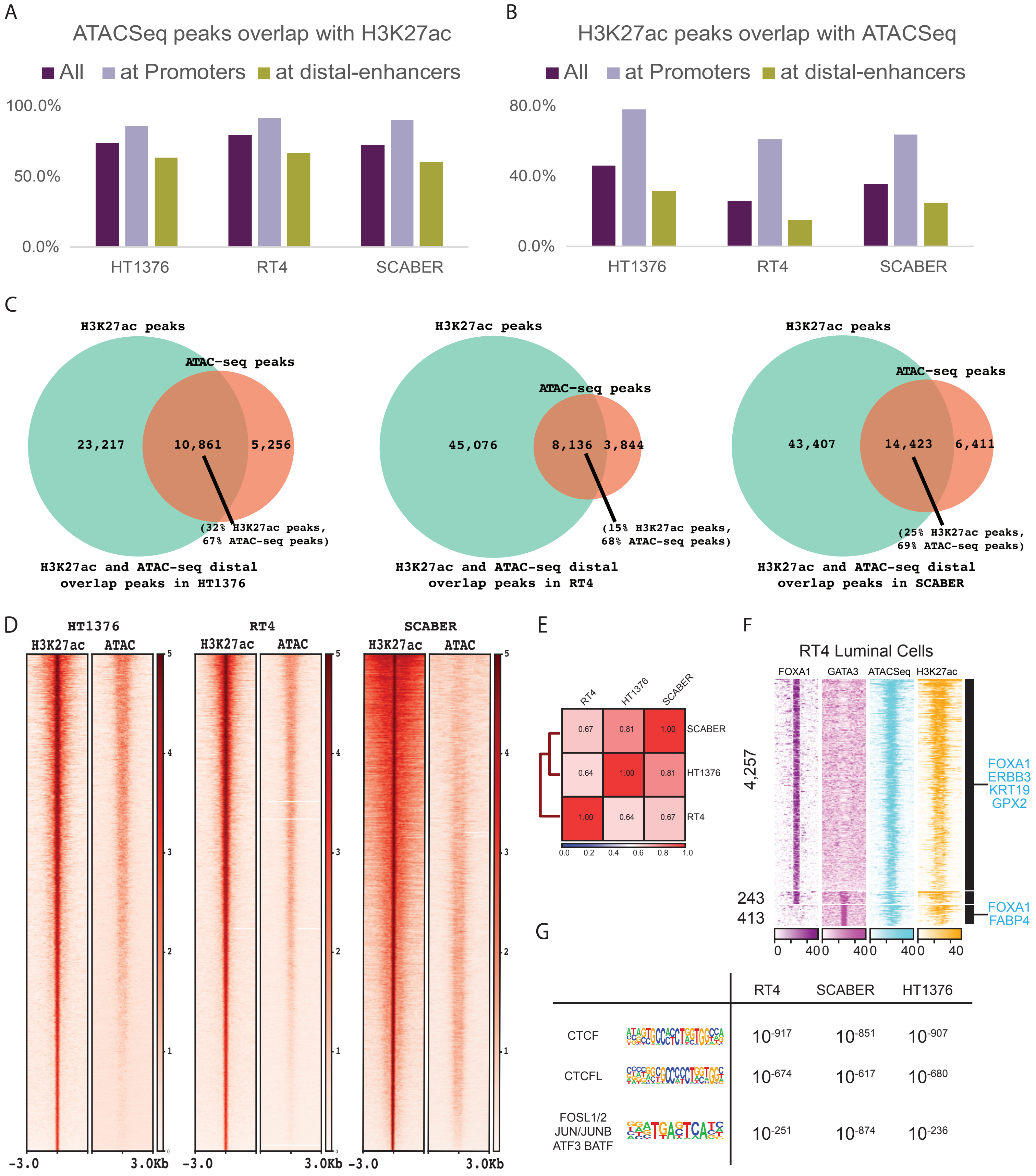

Figure S2: Epigenetic landscape analysis of open-chromatins in luminal and basal subtypes of bladder cancers. A) Genome-wide overlap of ATAC-Seq peaks with H3K27ac ChIP-Seq is shown here for each cell line at either promoter, enhancer or all locations. B) Genome-wide overlap of H3K27ac ChIP-Seq peaks with ATAC-Seq is shown here for each cell line at either promoter, enhancer or all locations. C) Overlap between distal H3K27ac and ATAC-seq peaks. D) ATAC-seq signal at distal enhancers compared with distal H3K27ac signal. E) Genome-wide correlation of ATAC-Seq signals between cell lines recapitulate enhancer/promoter and RNA-Seq based clustering. F) FOXA1 and GATA3 ChIP-Seq binding sites overlapped at promoters are shown here as genome-wide tag plot in three groups. G) A comparison of top 3 motifs enriched p-values in each open-chromatins that does not overlap with any H3K27ac signals within its cell lines are shown.

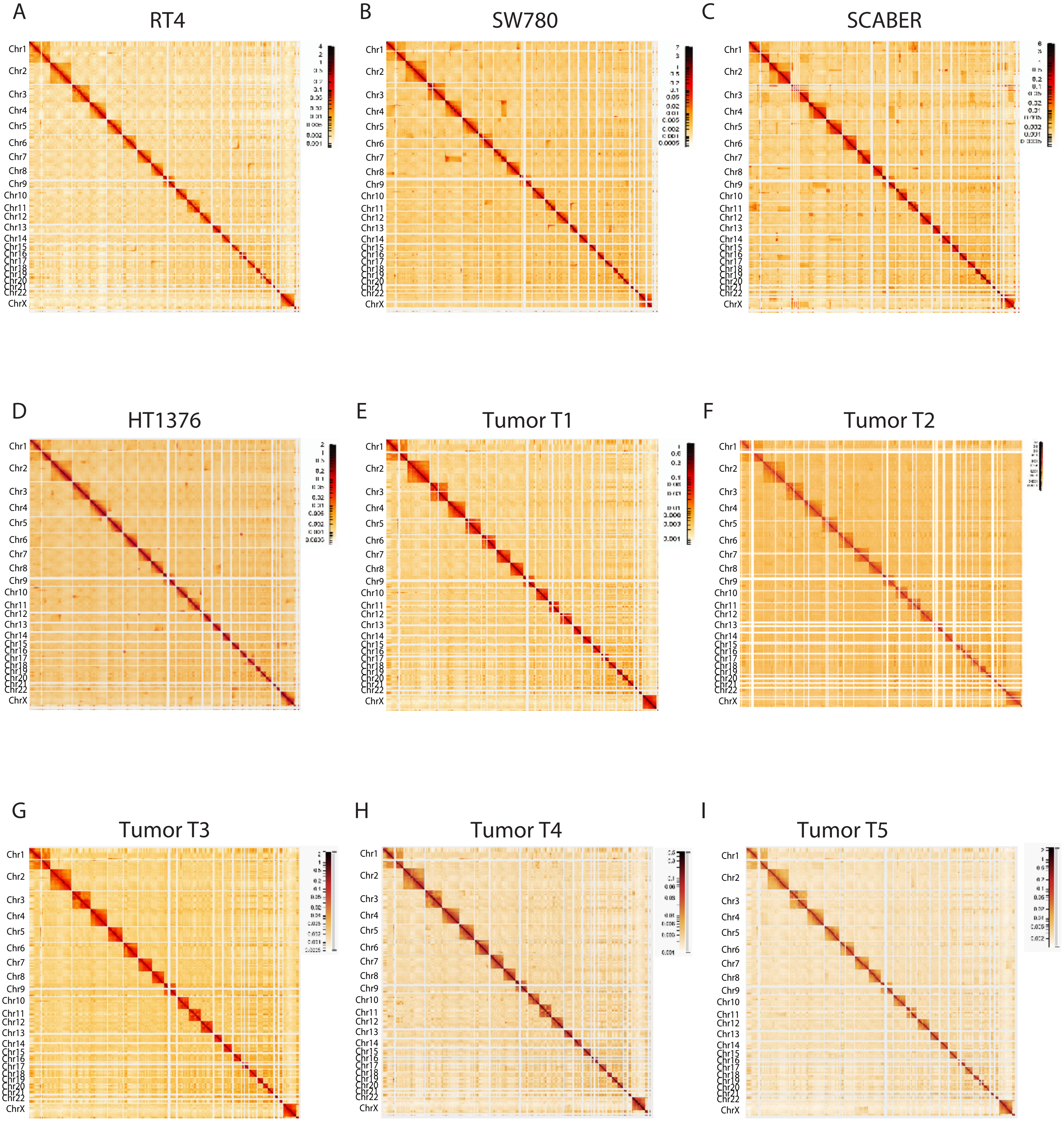

Figure S3: Hi-C maps of luminal and basal subtypes of bladder cancers and bladder tumors. Genome-wide chromosome view of Hi-C map is shown for RT4 (A), SW780 (B), SCABER (C), HT1376 (D), tumor T1 (E), tumor T2 (F), tumor T3 (G), tumor T4 (H) and tumor T5 (I) at 10MB resolution.

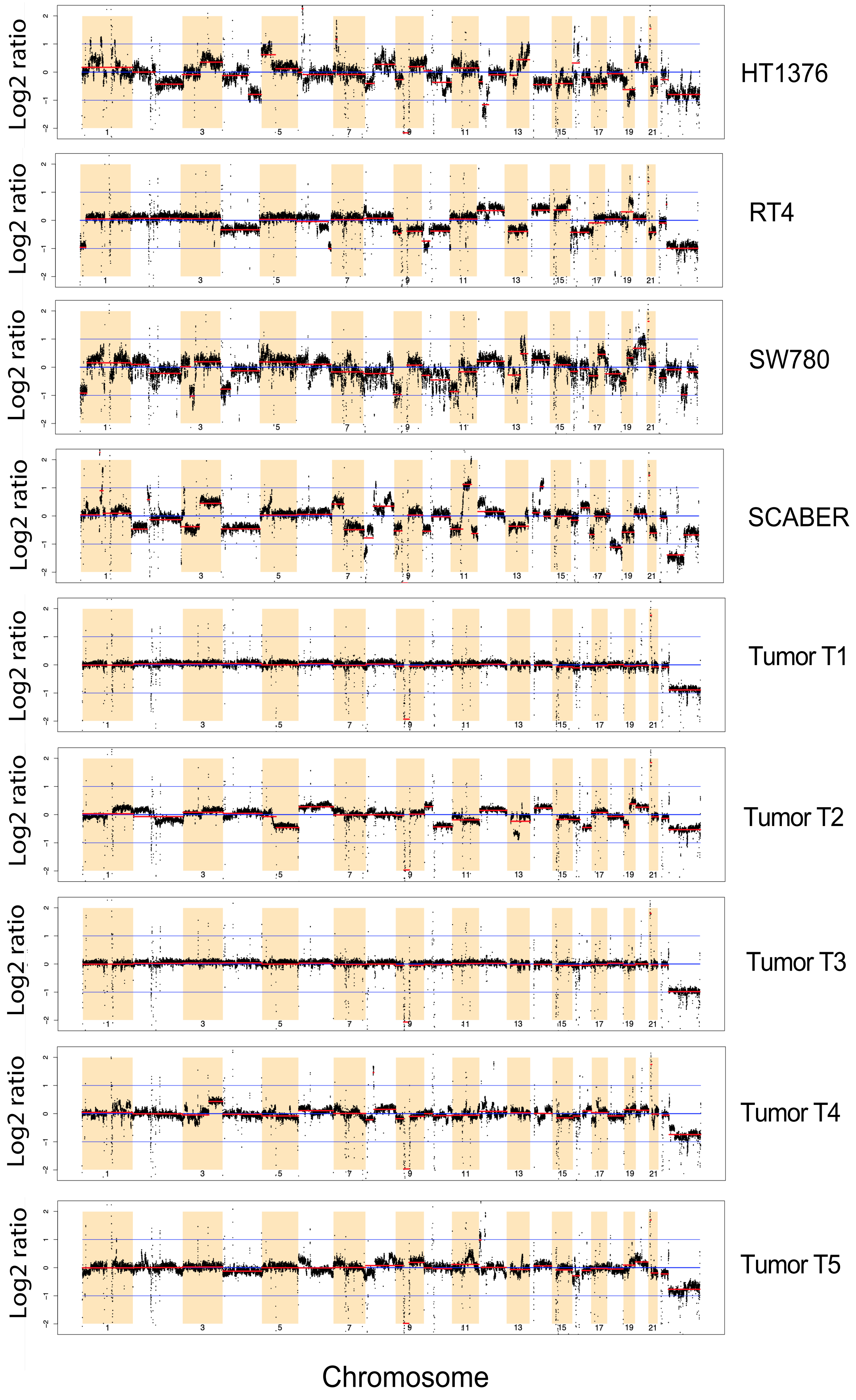

Figure S4: Copy number profiles for four bladder cancer cell lines(HT1376, RT4, SW780 and SCABER) and five tumor samples(Tumor T1, Tumor T2, Tumor T3, Tumor T4 and Tumor T5). CNVs were computed using Hi-C data.

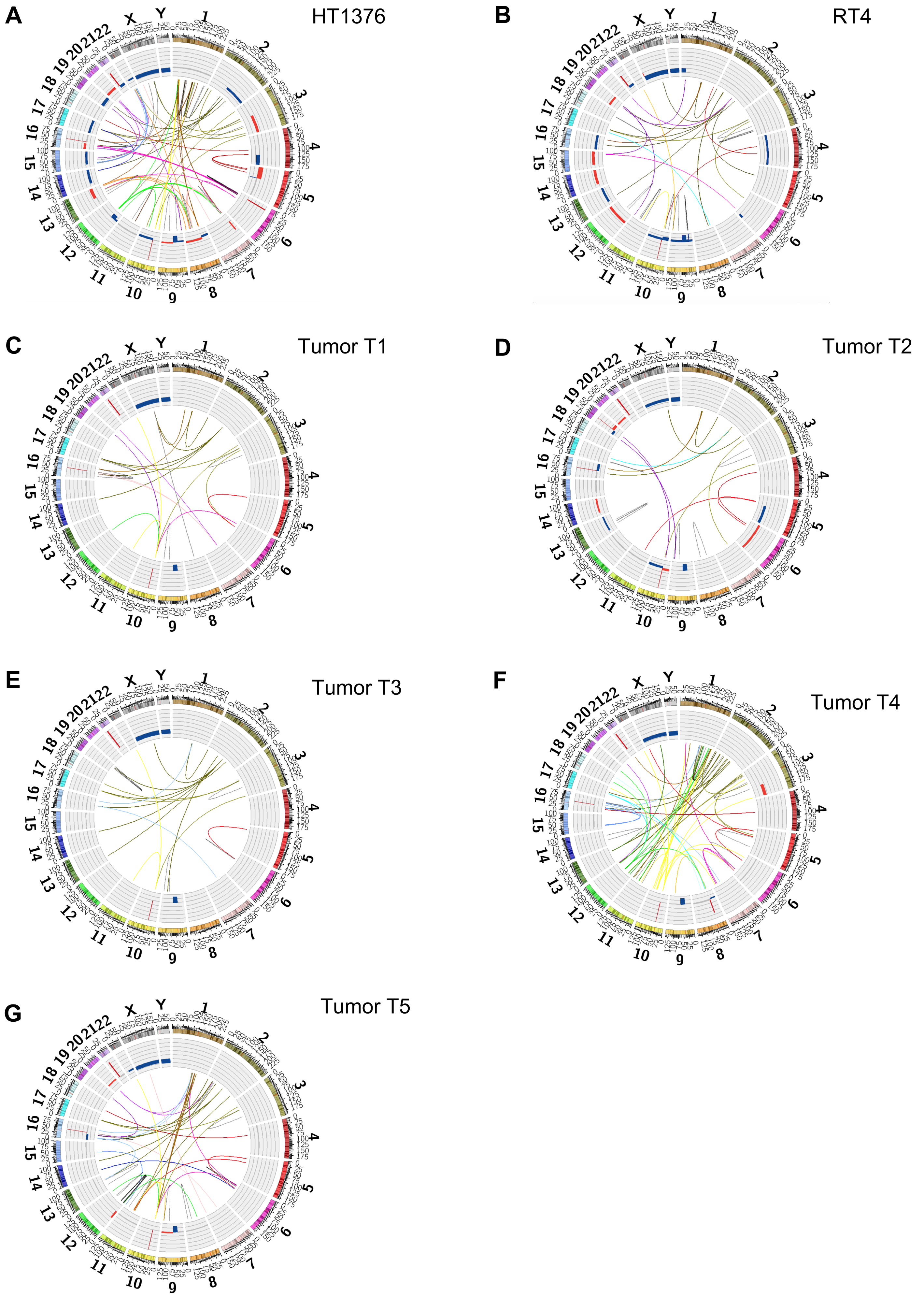

Figure S5: Intra- and inter-chromosome structure variation (SV) events. Circos plot showing intra- and inter-chromosome SVs in HT1376 (A), RT4 (B), Tumor T1 (C), Tumor T2 (D), Tumor T3 (E), Tumor T4 (F) and Tumor T5 (G).
