# Supplementary material for: Subtype-associated epigenomic landscape and 3D genome structure in bladder cancer
Source: Genome Biol. 2021 Apr 15;22:105. doi: 10.1186/s13059-021-02325-y (PMC8048365; doi:10.1186/s13059-021-02325-y)
Supplement: Supplementary file 16 — Additional file 16. Computational data analysis methods. [file 13059_2021_2325_MOESM16_ESM.docx]

**Computational methods**

**RNA-Seq analysis**

We used the standard ENCODE RNA-Seq pipeline (ENCODE consortium: https://github.com/ENCODE-DCC/rna-seq-pipeline) for mapping and calling gene and isoform level RSEM log2 counts. Briefly, FASTQ files were trimmed for adapters and mapped against hg19 human reference genome using STAR. Gene-level abundances of transcripts were estimated from RNA-Seq bam files using Kallisto program [1]. Gene-level and isoform-level abundances were also quantitated using RSEM program [2]. Then the QC was assessed by computing the median absolute deviation (MAD) between two replicates for non-zero FPKM values (normalized).

**ATAC-Seq analysis**

We used the standard ENCODE ATAC-Seq pipeline (ENCODE consortium: https://github.com/ENCODE-DCC/atac-seq-pipeline) for mapping and calling significant peaks for enrichment of transposase insertions. Briefly, raw FASTQ files were mapped against the hg19 human reference genome build. Adapters were trimmed and alignment was done using Bowtie2. Blacklisted chromosomes including ChrM reads were removed. Then aligned reads were sorted by chromosome order and duplicates were marked and removed. For peak calling we used MACS2 program to report enrichment of transposase insertion frequency. For genome-browser visualization of tracks, we used bigwig files that contains p-value enrichment (-log10(pvalue)) generated by MACS2 program.

**ChIP-Seq analysis**

We used the standard ENCODE RNA-Seq pipeline (ENCODE consortium: https://github.com/ENCODE-DCC/chip-seq-pipeline2) for mapping and calling significant peaks for enrichment of H3K27ac binding. Briefly, raw FASTQ files were mapped against the hg19 human reference genome build. Adapters were trimmed and alignment was done using Bowtie2. Blacklisted chromosomes including ChrM reads were removed. Then aligned reads were sorted by chromosome order and duplicates were marked and removed. For peak calling we used MACS2 program to report enrichment of H3K27ac/GATA3 peaks by using input control (no pull-down control). For genome-browser visualization of tracks, we used bigwig files that contains p-value enrichment (-log10(pvalue)) generated by MACS2 program.

**Hi-C analysis**

For HiC analysis, each library raw FASTQ files were mapped against hg19 human reference build genome using runHiC pipeline[3], which is based on 4DN consortium. Briefly, mapped FASTQ files were aligned using bwa aligner and reads were filtered for quality and PCR duplicates. Fragments were assembled based on pairs of reads mapped to long stretch of DNA and further filtered for fragments that contain ligation of at least 2 different restriction fragments. Self-ligated fragments were filtered out this fashion. Then the reads were binned at multiple resolution of data: 1KB, 5KB, 10KB, 25KB, 50KB, 100KB, 500KB, 1MB; to generate a contact matrix using cooler tools (*.mcool file). Quality of data was assessed by calculating the ratio of reads mapped at contact distances >20KB as usable read matrix.

**Differential expression of gene (DEG) analysis**

We used DEseq2[4] to identify the differential expressed genes between luminal (RT4 and SW780) and basal (SCABER and HT1376) cell lines using our RNA-seq data. RT4 and SW780 are considered replicates, so are SCABER and HT1376 when identifying luminal specific and basal specific genes. The cutoff we used is log2FoldChange > 2 and adjusted p-value < 0.01.

**Reads in Peaks normalization of ChIP-Seq and ATAC-Seq signals for construction of signal intensity matrix and plotting correlations**

Since the read depth/quality varied between different samples, a similar approach to Corces et al was used to transform each sample by reads-in-peaks normalization [5]. For each sample, its bam file and MAC2 peak file was used to calculate the total reads mapped in the MACS2 called peak region (RiP - reads in peaks). Then a scale factor was established for each sample to appropriately scale it to 10 million reads for the total number of reads mapped within called peak regions. Then the samples signals were scaled genome-wide using the bamCoverage tool from deepTools [6] to obtain a bigwig file. Then, multiBigwigSummary tool from deepTools was used for plotting genome-wide correlations between ATAC-Seq/H3K27ac samples. For obtaining H3K27ac or ATAC-Seq signal intensity matrix, multiBigwigSummary tool from deepTools was used by specifying an input BED-file of H3K27ac or ATAC-Seq clusters, respectively.

**Intersection of Bladder Cancer Cell line peaks (H3K27ac/ATAC-Seq) for obtaining relevant clusters**

Peaks were clustered manually by using BEDtools intersect function [7]. For luminal specific cluster, we obtained RT4 and SW780 shared peaks that are not found in other cell lines. For basal specific cluster, we obtained SCABER and HT1376 shared peaks that are not found in other cell lines. For shared cluster, we obtained peaks that are common to all 4 cell lines. Peaks located in these 3 clusters in each type of data (H3K27ac/ATAC-Seq) were further used for downstream analysis. To obtain distal open chromatin regions, peaks located within promoters were removed from ATAC-Seq clusters. Clusters for each dataset was visualized by using computeMatrix reference-point tool in deepTools followed by plotHeatmap tool.

**Peak-gene association analysis for H3K27ac data**

ChIP-Seq H3K27ac peaks from the above 3 clusters were separated as distal-enhancers or proximal-promoters based on the distance (-2KB, +2KB) of their location from transcription start site (TSS) of nearest gene. Peaks located within promoters were assigned to the gene for which the TSS belonged to. For distal-enhancer peaks, each peak was first linked to the top 20 (by distance) nearest genes located within ± 500KB. Then each peak signal intensities (log2 transformed) were used from the previously constructed signal intensity matrix to correlate to the putatively linked gene expression (log2 TPM). A background expected correlation model was constructed by using a random 10,000 peak-gene associations. Each peak-gene correlation was then scored for statistical significance from the random model and further assessed for false-discovery using Benjamini-Hochs method. Top variable distal-enhancer to gene associations were obtained by selecting top 10,000 variable signals that showed >0.5 correlation to gene expression values with <0.01 q-value filtering. Top variable promoter to gene plot was also prepared by selecting top 10,000 variable signals. Finally, heatmaps were plotted using ComplexHeatmap [8] R package to visualize peak-gene associations by plotting both the signal intensity values for H3K27ac as well as gene expression TPM values.

**ATAC-Seq and transcription factor (TF) motif analysis**

ATAC-Seq luminal-specific and basal-specific clusters obtained from the previous analysis was used to call motifs. ATAC-Seq peaks from clusters that are in distal regions (denoted as distal-enhancers) were used to conduct MOTIF search in distal-enhancer regions. Motifs were called using Homer package (http://homer.ucsd.edu/homer/index.html) [9] using their default settings. MOTIF results were summarized and visualized as ranked plot. Ranked motif plot was plotted by ranking motifs discovered by their P value (<0.01). FOXA1 and GATA3 ChIP-Seq peaks located at distal regions or at proximal promoter regions were also intersected with ATAC-Seq and H3K27ac ChIP-Seq datasets from RT4 cells. Peaks were then subsequently used as inputs for motif search and summarized.

**TCGA ATAC-Seq clustering plot**

ATAC-Seq data from TCGA bladder tumors was downloaded from Corces et al [5].Downloaded data signal files (hg38) were then cross-mapped to human build hg19. Signal intensity matrix for all TCGA bladder tumors along with our cell line ATAC-Seq data was constructed using the multiBigwigSummary tool from deepTools by specifying an input BED-file of H3K27ac clusters. We used ComBat batch effect removal tool to separate the variability that we may see due to the different processing methods and sequencing source. To assess the similarity of our cell lines with TCGA bladder tumor samples, correlation heatmaps were plotted and visualized using R ComplexHeatmap package.

**Peakachu loop calling and analysis**

Hi-C cooler (*.mcool) files were visualized using HiGlass [10]package. Peakachu loop caller was used to call loops for each sample at 10KB resolution of Hi-C data [11]using score_genome function by using pretrained models (both CTCF and H3K27ac at 10% or 20%, depending on total intra reads number) from GM12878. Loops were then pooled by using peakachu pool function with a probability cutoff of 0.8. Luminal specific loops are defined as ICE signal larger than 0.005 in both RT4 and SW780 cell lines, while basal specific loops are defined as ICE signal larger than 0.005 in both SCABER and HT1376 cell lines. Aggregated plot analysis (APA) was plotted using apa-analysis tool from HiCPeaks[12]. Luminal-specific and basal-specific loops were intersected using pairToPair BEDTools function, to report the observed loops in tumors samples. Loops were visualized as bedpe file format using integrated genome browser (IGV) [13].

For characterizing the types of loops, enhancers and promoters were defined using H3K27ac and H3K4me3 ChIP-Seq peaks, respectively. Then we counted the ratio of loops in each cell line with different regulatory element combinations at anchor loci. For example, a loop is determined as an enhancer-promoter loop if there are at least one enhancer located within ± 20Kb of one of its anchors, and at least one promoter located within ± 20Kb of another anchor.

FOXA1 and GATA3 enrichment at loop anchors were computed. First, 10Kb bins were extracted for +/- 300Kb region of each non-redundant loop anchor. Then the number of FOXA1/GATA3 ChIP-Seq peaks were counted in each bin. Finally, the number of peaks were averaged across all loop anchors for each bin.

**Structural variations analysis**

Cnv function of HiNT [14] and hic-breakfinder[15] were used on HiC data to infer CNV and identify structural variations (including deletions, inversions, and translocations), respectively. Previously generated .hic files and bam files were used as input. Our in-house pipeline (under review) were used to identify neo-loops or enhancer hijacking events. Genomic locations were re-assembled based on the breakpoints identified by hic-breakfinder, and then loops were called using peakachu at the re-assembled regions.

**NPAS2 Kaplan-Meier plot**

R package survival was used to analyze and plot survival analysis for TCGA patients[16]. Gene expression TPM (log2 and mean-normalized) values from TCGAbiolinks were used for separating patients with high-expressing (log2(FC to mean value) ≥ 1) and low-expressing (log2(FC to mean value) ≤ 1) NPAS2. Function survfit() was used to plot survival curve. Log rank and Cox-hazard tests were computed by using survdiff() and coxph() functions, respectively.

**References**

1. Bray NL, Pimentel H, Melsted P, Pachter L: **Near-optimal probabilistic RNA-seq quantification.** *Nat Biotechnol* 2016, **34:**525-527.

2. Li B, Dewey CN: **RSEM: accurate transcript quantification from RNA-Seq data with or without a reference genome.** *BMC Bioinformatics* 2011, **12:**323.

3. Wang X: **runHiC: A user-friendly Hi-C data processing software based on hiclib.** *Zenodo* [*http://doiorg/105281/zenodo55324*](http://doiorg/105281/zenodo55324) 2016.

4. Love MI, Huber W, Anders S: **Moderated estimation of fold change and dispersion for RNA-seq data with DESeq2.** *Genome Biol* 2014, **15:**550.

5. Corces MR, Granja JM, Shams S, Louie BH, Seoane JA, Zhou W, Silva TC, Groeneveld C, Wong CK, Cho SW, et al: **The chromatin accessibility landscape of primary human cancers.** *Science* 2018, **362**.

6. Ramirez F, Dundar F, Diehl S, Gruning BA, Manke T: **deepTools: a flexible platform for exploring deep-sequencing data.** *Nucleic Acids Res* 2014, **42:**W187-191.

7. Quinlan AR, Hall IM: **BEDTools: a flexible suite of utilities for comparing genomic features.** *Bioinformatics* 2010, **26:**841-842.

8. Gu Z, Eils R, Schlesner M: **Complex heatmaps reveal patterns and correlations in multidimensional genomic data.** *Bioinformatics* 2016, **32:**2847-2849.

9. Heinz S, Benner C, Spann N, Bertolino E, Lin YC, Laslo P, Cheng JX, Murre C, Singh H, Glass CK: **Simple combinations of lineage-determining transcription factors prime cis-regulatory elements required for macrophage and B cell identities.** *Mol Cell* 2010, **38:**576-589.

10. Kerpedjiev P, Abdennur N, Lekschas F, McCallum C, Dinkla K, Strobelt H, Luber JM, Ouellette SB, Azhir A, Kumar N, et al: **HiGlass: web-based visual exploration and analysis of genome interaction maps.** *Genome Biol* 2018, **19:**125.

11. Salameh TJ, Wang X, Song F, Zhang B, Wright SM, Khunsriraksakul C, Ruan Y, Yue F: **A supervised learning framework for chromatin loop detection in genome-wide contact maps.** *Nat Commun* 2020, **11:**3428.

12. Rao SS, Huntley MH, Durand NC, Stamenova EK, Bochkov ID, Robinson JT, Sanborn AL, Machol I, Omer AD, Lander ES, Aiden EL: **A 3D map of the human genome at kilobase resolution reveals principles of chromatin looping.** *Cell* 2014, **159:**1665-1680.

13. Thorvaldsdottir H, Robinson JT, Mesirov JP: **Integrative Genomics Viewer (IGV): high-performance genomics data visualization and exploration.** *Brief Bioinform* 2013, **14:**178-192.

14. Wang S, Lee S, Chu C, Jain D, Kerpedjiev P, Nelson GM, Walsh JM, Alver BH, Park PJ: **HiNT: a computational method for detecting copy number variations and translocations from Hi-C data.** *Genome Biol* 2020, **21:**73.

15. Dixon JR, Xu J, Dileep V, Zhan Y, Song F, Le VT, Yardimci GG, Chakraborty A, Bann DV, Wang Y, et al: **Integrative detection and analysis of structural variation in cancer genomes.** *Nat Genet* 2018, **50:**1388-1398.

16. Meszaros G, Solkner J, Ducrocq V: **The Survival Kit: software to analyze survival data including possibly correlated random effects.** *Comput Methods Programs Biomed* 2013, **110:**503-510.
