# Supplementary material for: Subtype-associated epigenomic landscape and 3D genome structure in bladder cancer
Source: Genome Biol. 2021 Apr 15;22:105. doi: 10.1186/s13059-021-02325-y (PMC8048365; doi:10.1186/s13059-021-02325-y)
Supplement: Supplementary file 17 — Additional file 17. Review history. [file 13059_2021_2325_MOESM17_ESM.docx]

Review History

**First round of review**

**Reviewer 1**

**Are you able to assess all statistics in the manuscript, including the appropriateness of statistical tests used?** Yes, and I have assessed the statistics in my report.

**Comments to author:**

The authors performed RNA-seq, ATAC-seq, ChIP-seq for H3K27ac, and Hi-C on two luminal, two basal bladder cancer cell lines, and two patient derived muscle-invasive bladder tumors. They compared their RNA-seq with publicly available datasets to confirm their subtype classification and differential gene expression analysis identified genes upregulated in basal (enriched for pathways including in ECM organization, leukocyte activation, cytokine production) or luminal (enriched for pathways including metabolism and catabolism of lipids) cells. H3K27ac was used to identify active promoters and enhancers. These signals were indicative of luminal or basal gene expression. The difference in enriched peaks in tumor 2 compared with tumor 1 is somewhat concerning. Is this due to quality of ChIP? ATACseq identifying open chromatin demonstrated a significant overlap with H3K27ac particularly within promoters. What accounts for the lower overlap in distal regions? Motif analysis identified TAD1, KLF and TFAP2C in basal open chromatin. AP-1 complex proteins were the top most enriched in both luminal and basal open chromatin regions. Thyroid hormone receptor was identified as a motif not previously implicated in bladder cancer. They determined that forkhead and GATA motifs were present in open chromatin in all of the subtypes which is surprising considering FOXA1 is low in basal cells. Stem cell associated pioneering TFs such as KLF10/14 and NANOG were identified in basal bladder enhancers which is interesting given a progenitor subpopulation within these cells. Despite this, motif analysis must be viewed skeptically in general and requires much more work to determine if these factors are in actuality present. For example, multiple TFs can bind to very similar motifs. Also, this points out that all of these analyses are population averages and include the multiple subpopulations of cells that exist within the cell lines. This is particularly an issue within tumors which have considerable heterogeneity. Furthermore, copy number variation, inversions, and deletions can have profound impacts on higher order chromatin organization. Do the authors have any indication of these events in their cell lines/ tumors? As mentioned above it is important to validate motif analyses and this work went on to perform GATA3 and FOXA1 ChIP-seqs. The authors identified luminal and basal specific loops in Hi-C analyses. Within tumor samples, the authors show 10-20% of these luminal or basal specific loops are found in the tumor samples. This seems like a low amount of concordance. Are these loops found with a lower frequency to any tumors of different cells of origin in publicly available datasets? Based upon their genome-wide datasets the authors identified NPAS2 as a novel luminal bladder cancer gene involved in cell migration. This is a particular strength of the paper as they then functionally validate this gene. Overall this work is interesting, novel and important to the field and should be considered for publication with major revision.

**Reviewer 2**

**Are you able to assess all statistics in the manuscript, including the appropriateness of statistical tests used?** No, I do not feel adequately qualified to assess the statistics.

**Comments to author:**

Iyyanki et al performed multiplatform sequencing analysis on 4 bladder cancer cell lines and two primary tumors to evaluate an open chromatin mark H3K27ac and enhancer landscape revolving FOXA1 and GATA3 transcription in luminal bladder cancer. This is an interesting study describing multiomics analysis. However, many data description and interpretation does not reflect original raw data, which confused the reviewer and readers. Specific comments:

1. The authors clustered bladder tumors into four groups/clusters, claiming to be consistent with previous studies; however, it is unclear i) how they correspond with the TCGA clusters I-IV? and ii) how the Luminal-I and Liminal-II are related to the luminal-papillary, luminal-infiltrated and luminal indicated by TCGA 2017?
   a. Exactly what are the four CLUS in Figure 1C? There are no descriptions (when they talked about clusters, people immediately thought they are referring to TCGA 4 clusters, but they aren’t)
   b. No indications why they showed Chr14 in Figure 1B and what’s the significance? Why FOXA1, a luminal driver, for SCABER (basal) is more similar to luminal (H3K7ac) than HT1376 (luminal-basal)?
2. Page 8, they claimed HT1376 showed similar pattern to Luminal? How? (Fig. 2C)

3. Page 8, “… luminal genes were predominately shared between luminal and luminal-basal …?” How? (Fig. 2E) … extremely confusing what they are referring to?

4. The authors made big claims about distinction between promoter-enhancer loops between basal and luminal tumors (or luminal-basal), however, based on their figures, we cannot see the type of distinction they claimed (e.g. Figure 4E, Figure S5). They might need to label their figures more intentionally to pinpoint exactly what changes they are referring to.
5. A) It is unclear why the authors keep changing their clustering methodology e.g. Figure 1a versus Figure 5b, even using the same TCGA cohort, and changed their color scheme for their clustering with no description. B) It is unclear why they pick NPAS2 from their venn diagram Figure 5A, a gene that is shared between basal and luminal and then claim that it is luminal-specific? (it is unclear what the colors meant? It is very frustrating to read and try to find information throughout)
6. When one looks at the function of TFs, we highly recommend analyzing them in the context of the cell line within the same subtype, i.e. knockout the TFs in luminal cells and see how they impact other TF binding to the expected sites via Chip-PCR and see if they compensate for each other? It is unclear why they overexpress a luminal TF NPAS2 in a basal model?
7. And why would they use cell migration as an experimental endpoint (Figure 5H)? If they claim that overexpression of NPAS2 convert a basal into luminal phenotype, should they utilize a differentiation assay or at least look at the panel of basal/luminal markers (via qPCR)?
8. What’s the clinical implication by showing one luminal TF regulate expression of another luminal TF?

**Authors Response**

**Point-by-point responses to the reviewers’ comments:**

***Reviewer #1 (Remarks to the Author):***

*Summary:*

*The authors performed RNA-seq, ATAC-seq, ChIP-seq for H3K27ac, and Hi-C on two luminal, two basal bladder cancer cell lines, and two patient derived muscle-invasive bladder tumors. They compared their RNA-seq with publicly available datasets to confirm their subtype classification and differential gene expression analysis identified genes upregulated in basal (enriched for pathways including in ECM organization, leukocyte activation, cytokine production) or luminal (enriched for pathways including metabolism and catabolism of lipids) cells. H3K27ac was used to identify active promoters and enhancers. These signals were indicative of luminal or basal gene expression.*

*Motif analysis identified TAD1, KLF and TFAP2C in basal open chromatin. AP-1 complex proteins were the top most enriched in both luminal and basal open chromatin regions. Thyroid hormone receptor was identified as a motif not previously implicated in bladder cancer. They determined that forkhead and GATA motifs were present in open chromatin in all of the subtypes which is surprising considering FOXA1 is low in basal cells. Stem cell associated pioneering TFs such as KLF10/14 and NANOG were identified in basal bladder enhancers which is interesting given a progenitor subpopulation within these cells.*

*Despite this, motif analysis must be viewed skeptically in general and requires much more work to determine if these factors are in actuality present. For example, multiple TFs can bind to very similar motifs. Also, this points out that all of these analyses are population averages and include the multiple subpopulations of cells that exist within the cell lines. This is particularly an issue within tumors which have considerable heterogeneity.*

*As mentioned above it is important to validate motif analyses and this work went on to perform GATA3 and FOXA1 ChIP-seqs. Based upon their genome-wide datasets the authors identified NPAS2 as a novel luminal bladder cancer gene involved in cell migration. This is a particular strength of the paper as they then functionally validate this gene. Overall this work is interesting, novel and important to the field and should be considered for publication with major revision.*

We thank the reviewer for the accurate summary of this work. We are also delighted that the reviewer found this work interesting and novel. Below we provide point-to-point responses to this reviewer’s suggestions.

*Specific comments:*

*1/ H3K27ac was used to identify active promoters and enhancers. These signals were indicative of luminal or basal gene expression. The difference in enriched peaks in tumor 2 compared with tumor 1 is somewhat concerning. Is this due to quality of ChIP?*

We agree with the reviewer, and we have also identified the problem – sample T2 has been stored in the freezer for many years, which raises some concerns in regards to the quality of this sample. For T2, we believe this potential sample quality issue may explain why RNA-Seq and ChIP-Seq data look different. Therefore, we decided to exclude the ChIP-Seq and RNA-seq for T2 and collected tissue from three new bladder cancer patients and performed RNA-Seq, ChIP-Seq, and Hi-C experiments on the fresh samples.

The statistics for the ChIP-Seq data in three new tumor samples are listed below:

**Table 1. Statistics of ChIP-Seq data in the three new tumor samples**

| New tumor sample id | Raw read counts | Mapped reads | Uniquely mapped reads | Uniquely mapped ratio | Num. of Peaks |
| --- | --- | --- | --- | --- | --- |
| T3 | 66,327,706 | 62,344,213 | 40,379,348 | 0.61 | 91,786 |
| T4 | 45,921,160 | 41,923,901 | 28,610,212 | 0.62 | 66,683 |
| T5 | 48,603,530 | 43,983,675 | 26,136,840 | 0.54 | 84,405 |

We have incorporated these three additional tumor samples into the clustering analysis as tumor T3, tumor T4 and tumor T5. The revised Figure 1G shows that all the three additional tumor samples show high correlation with the original tumor T1.

**New Figure 1G. Clustering analysis of the H3K27ac signals across bladder cancer cell lines and primary tissues.**

*2/* *ATACseq identifying open chromatin demonstrated a significant overlap with H3K27ac particularly within promoters. What accounts for the lower overlap in distal regions?*

We thank the reviewer for raising this question. We believe the main reason is that the total number of ATAC-Seq peaks and H3K27ac peaks is different. As shown below, using the ENCODE pipeline, the number of reproducible peaks we identified in ATAC-Seq is 20-30k, while there are ~50-80k H3K27ac peaks in each cell type. Therefore, only a small percentage of distal H3K27ac peaks overlap with ATAC-Seq peaks. Concurrently, 67-69% of the distal ATAC-Seq peaks overlap with H3K27ac peaks.

**Supplementary Figure 2C. Overlap between distal H3K27ac and ATAC-seq peaks.**

To investigate whether the stringent peaking calling contributes to the smaller percentage of the overlap, we performed the following analysis. We ranked all distal H3K27ac peaks according to the MACS probability scores in all three samples, then plotted the ATAC-seq signals at these sites (Supplementary Fig. 2D, listed below). We observed that the majority of the distal H3K27ac enhancers show enrichment of ATAC-Seq signals.

**Supplementary Figure 2D. Comparing ATAC-seq signal with distal H3K27ac distal enhancers.**

*3/ Furthermore, copy number variation, inversions, and deletions can have profound impacts on higher order chromatin organization. Do the authors have any indication of these events in their cell lines/ tumors?*

We thank the reviewer for this expert suggestion, and we indeed observed such events. We first systematically identified copy number variations (CNVs) and SV events using the Hi-C data with the HiNT [2] and the Hi-Cbreakfinder [3] software that we previously co-developed. As shown below, we observed extensive CNVs in all the cancer cell lines.

**Supplementary Figure 4. Copy number profiles calculated by HiNT for each bladder cancer cell line and primary tumor sample.**

Below, we summarized the number of translocations and large SVs we identified in this study.

**Supplementary Table 6. Num. of large SVs in bladder cancer cell lines and primary tumor samples**

| **Cell line/patient** | **Inversion (>1Mb)** | **Deletion (>1Mb)** | **Translocation** |
| --- | --- | --- | --- |
| HT1376 | 8 | 8 | 73 |
| RT4 | 3 | 8 | 30 |
| SW780 | 30 | 17 | 27 |
| SCABER | 17 | 7 | 33 |
| Tumor T1 | 3 | 2 | 22 |
| Tumor T2 | 2 | 4 | 14 |
| Tumor T3 | 3 | 3 | 18 |
| Tumor T4 | 5 | 6 | 59 |
| Tumor T5 | 8 | 5 | 39 |

To investigate the potential impact of these genomic rearrangements on higher order chromatin organization, we used an in-house pipeline to reconstruct the local Hi-C map surrounding the breakpoints of the re-arranged regions. A few selected examples are shown below, where the large SVs or inter-chromosomal translocations might induce cancer specific chromatin interaction. Consistent with previous studies [4], different kind of SVs often cause neo-TAD and neo-loop formation, which might further contribute to the dysregulation of gene expression in bladder cancer. Therefore, we believe the Hi-C maps generated in this study present great value in the study of gene regulation events for the bladder cancer community.

**New Figure 4: Chromatin interactions induced by structure variation (SV) events.** A-B) Circos plot showing intra- and inter-chromosome SVs in SCABER (A) and SW780 (B). **C**) A large intra-chromosomal translocation on chr9. D-H) Inter-chromosomal translocations. The breakpoints were identified by the HiCBreakfinder software. We then reconstructed the local Hi-C maps across the breakpoints. RNA-Seq and H3K27ac ChIP-Seq tracks from the same cell type are shown below the Hi-C maps.

*4/. The authors identified luminal and basal specific loops in Hi-C analyses. Within tumor samples, the authors show 10-20% of these luminal or basal specific loops are found in the tumor samples. This seems like a low amount of concordance. Are these loops found with a lower frequency to any tumors of different cells of origin in publicly available datasets?*

We thank the reviewer for this observation. The low concordance is due to the low sequencing depths, which led to low-resolution Hi-C loops. Therefore, during the revision, we sequenced the Hi-C libraries much more deeply in four cell lines (RT4, SW780, SCABER, and HT1376) and reached ~ 1 billion reads for each sample. In addition, we also performed Hi-C on **three new primary tumor samples (T3, T4, and T5)**. In total, we generated over 7 billion reads of Hi-C data on which to perform high-resolution chromatin loop analysis. The statistics for all 9 samples are shown below:

**Table 2 Hi-C statistics of four cell lines and five tumor samples**

| Cell line/patient | Total read pairs | Mapped pairs | No dup pairs | Trans interaction | Ratio of trans | Cis>20KB | Loop no. |
| --- | --- | --- | --- | --- | --- | --- | --- |
| RT4 | 1,110,324,569 | 763,324,521 | 665,902,485 | 168,445,665 | 25.30% | 371,405,839 | 51,836 |
| SW780 | 1,006,054,492 | 719,950,959 | 601,562,504 | 50,396,989 | 8.38% | 297,911,053 | 66,121 |
| SCABER | 1,365,004,324 | 1,038,868,191 | 811,466,489 | 68,522,272 | 8.44% | 478,744,593 | 69,032 |
| HT1376 | 872,959,317 | 563,740,916 | 487,583,546 | 110,096,445 | 22.58% | 252,724,800 | 38,271 |
| Tumor T1 | 1,142,095,420 | 819,487,795 | 687,380,365 | 181,412,008 | 26.39% | 340,453,099 | 38,268 |
| Tumor T2 | 910,530,839 | 648,347,387 | 558,499,961 | 205,505,306 | 36.80% | 259,381,419 | 27,806 |
| Tumor T3 | 889,173,301 | 647,861,249 | 506,642,279 | 144,396,463 | 28.50% | 273,351,824 | 32,958 |
| Tumor T4 | 1,035,354,320 | 734,508,740 | 567,804,336 | 223,415,935 | 39.35% | 256,314,526 | 21,162 |
| Tumor T5 | 1,046,420,046 | 749,749,120 | 585,067,931 | 201,865,160 | 34.50% | 272,879,971 | 35,111 |

With the high-resolution Hi-C data, we re-analyzed the Hi-C data of the four cell lines at 10kb resolution and **redefined the luminal- and basal- specific loops.** In total, we predicted 2,299 luminal-specific loops (RT4 and SW780 cells), and 2,144 basal-specific loops (SCABER and HT1376 cells). We then compared these newly defined subtype-specific loops with the five primary tumor samples (shown in Figure 3B), using the *bedtools pairtopair* function with *-slop* parameter setting to 25kb. We observed that the percentage of overlap between cell lines and clinical samples is increased, and is usually between 30-50%. In additional, tumors contain many additional cell types (fibroblasts, nerves, immune cells, etc.), which will lead to certain difference in loops called in cell lines and primary samples.

**Figure 3B. Contacts identified in luminal and basal-squamous cell lines are shared and validated in muscle-invasive bladder tumor samples.**

***Reviewer #2 (Remarks to the Author):***

*Summary:*

*Iyyanki et al performed multiplatform sequencing analysis on 4 bladder cancer cell lines and two primary tumors to evaluate an open chromatin mark H3K27ac and enhancer landscape revolving FOXA1 and GATA3 transcription in luminal bladder cancer. This is an interesting study describing multiomics analysis. However, many data description and interpretation does not reflect original raw data, which confused the reviewer and readers.*

We thank the reviewer for suggesting that this study is interesting. We apologize that some of the analysis in our original submission was not clear. We have made significant modifications in the result and discussion sections according to this reviewer’s suggestions.

*Specific comments:*

*1/* *The authors clustered bladder tumors into four groups/clusters, claiming to be consistent with previous studies; however, it is unclear i) how they correspond with the TCGA clusters I-IV? and ii) how the Luminal-I and Liminal-II are related to the luminal-papillary, luminal-infiltrated and luminal indicated by TCGA 2017?*

We thank the reviewer for this important suggestion on aligning the subtypes in different studies. We wanted to clarify that main goal of the clustering analysis is to identify bladder cancer cell lines that are representative of different subtypes. Therefore, we decided to apply the consensus MIBC subtyping methodology specified by Kamoun et al. Eur Urol. 2020[1]. Specifically, these authors compiled 1,750 MIBC transcriptomic profiles from 16 published datasets and two additional cohorts. More importantly, the authors also provided a R script to identify bladder cancer subtypes (<https://github.com/cit-bioinfo/consensusMIBC>). For this revised submission, we used their software to analyze the RNA-seq samples in this study and the results are listed below. This analysis confirmed that RT4 and SW780 are of the Luminal papillary subtype, while HT1376 and SCABER are of the Basal/squamous subtype. **Given this result, we removed the original Fig. 1B and instead present the following table.** We have also described their subtype in the main manuscript.

**Supplementary Table 1. Classification results for each sample using consensusMIBC**

| **sample** | **Class** | **P Value** |
| --- | --- | --- |
| RT4_rep1 | Luminal papillary | 3.05E-66 |
| RT4_rep2 | Luminal papillary | 2.22E-57 |
| SW780_rep1 | Luminal papillary | 5.31E-58 |
| SW780_rep2 | Luminal papillary | 6.18E-61 |
| HT1376_rep1 | Basal/squamous | 4.24E-47 |
| HT1376_rep2 | Basal/squamous | 1.53E-48 |
| SCABER_rep1 | Basal/squamous | 6.70E-48 |
| SCABER_rep2 | Basal/squamous | 7.49E-43 |
| Tumor1 | Luminal papillary | 1.13E-126 |
| Tumor3 | Stroma-rich | 4.46E-66 |
| Tumor4 | Basal/squamous | 1.41E-94 |
| Tumor5 | Basal/squamous | 1.76E-75 |

*1a/* *Exactly what are the four CLUS in Figure 1C? There are no descriptions (when they talked about clusters, people immediately thought they are referring to TCGA 4 clusters, but they aren’t)*

As mentioned above, we have switched to the method specified in Kamoun et al. [1] and have removed the clustering analysis.

*1b/* *No indications why they showed Chr14 in Figure 1B and what’s the significance? Why FOXA1, a luminal driver, for SCABER (basal) is more similar to luminal (H3K7ac) than HT1376 (luminal-basal)?*

As is common in large scale genomics studies, the main goal of the original Figure 1B was to provide the readers an overview of the quality of the genomic data generated in this study, such as Hi-C, ATAC-Seq, and ChIP-Seq data.

As for FOXA1, we apologize for the confusion. Because we showed a large region in Fig. 1B (7Mb region), it appears that the H3K27ac patterns in SCABER near FoxA1 look similar to the tracks in RT4 and SW780. However, when we zoom in on the FOXA1 gene (current Fig. 1D, below), we observe it is only active in luminal cells (RT4, SW780) but not in basal cells (SCABER, HT1376).

**Current Figure 1D.** Genome browser signal tracks for luminal genes FOXA1, CYP2J2 and basal genes KRT6A, KRT5, and KRT14 incorporating H3K27ac, ATAC-seq, and RNA-seq data collected from RT4, SW780, SCaBER, and HT1376 are shown.

*2/* *Page 8, they claimed HT1376 showed similar pattern to Luminal? How? (Fig. 2C)*

We apologize for the confusion. What we wanted to show is that, although based on global gene expression patterns, HT1376 is assigned as Basal/squamous; several luminal markers genes (such as KRT8 and GATA3) are also expressed in this cell type. We observed promoter mark at these genes as well.

We have re-written this sentence as “… *although HT1376 is classified as the* Basal/squamous *subtype, we observed that several luminal marker genes (such as KRT7/8 and GATA3) are expressed in this cell line and that their promoters also have H3K27ac signals.*”

*3/* *Page 8, “… luminal genes were predominately shared between luminal and luminal-basal …?” How? (Fig. 2E) … extremely confusing what they are referring to?*

We apologize for the confusion. In the first submission, as we observed that HT1376 expressed both luminal and basal marker genes, we referred this cell line as “luminal-basal.” In this revised manuscript, we examined its subtype using the new R script provided by Kamoun et al, and based on this approach, HT1376 is now defined as Basal/squamous. We have resolved the confusion and have re-written the sentence as follows:

*“… We observed that the enhancers show clea*r *clustering acco*rding to different cell types, and their target genes show similar *cell-type specific patterns (Fig. 1F and Fig. S1-D).”*

*4/* *The authors made big claims about distinction between promoter-enhancer loops between basal and luminal tumors (or luminal-basal), however, based on their figures, we cannot see the type of distinction they claimed (e.g. Figure 4E, Figure S5).* ***They might need to label their figures more intentionally to pinpoint exactly what changes they are referring to****.*

We thank the reviewer for this great suggestion. We’ve now modified the track panel name to make the figure clearer. The top arc tracks represent the enhancer-promoter loops in the four bladder cancer cell lines. Enhancer-promoter loops in luminal cell lines (RT4, SW780) are labeled in green and basal-specific EP loops (SCABER, HT1376) are labeled in purple.

**New Figure 3C.** Selected examples of subtype-specific enhancer-promoter loops.

*5a/ It is unclear why the authors keep changing their clustering methodology e.g. Figure 1a versus Figure 5b, even using the same TCGA cohort, and changed their color scheme for their clustering with no description.*

We apologize for the confusion. The reviewer is correct - we used an inconsistent color scheme in the annotation tracks between the original Fig. 1a and 5b.

As explained earlier, we have decided to remove our own clustering and have switched to the Kamoun et al. Eur Urol. 2020 [1]method. Therefore, we removed this 5b as well.

*5b/ It is unclear why they pick NPAS2 from their venn diagram Figure 5A, a gene that is shared between basal and luminal and then claim that it is luminal-specific? (it is unclear what the colors meant? It is very frustrating to read and try to find information throughout)*

We apologize for not demonstrating clearly why we chose NPAS2 to further investigate in this project. We have performed new analysis and below is why we think NPAS2 might be an important regulator in bladder cancer:

1) The reviewer is correct that the NPAS2 motif is enriched at both luminal-specific and shared ATAC-Seq peaks. However, we didn’t make it clear in the original submission that the significance of enrichment is different: P < 0.0001 in luminal-specific, and P < 0.001 in shared peaks.
2) When we examined the TF motifs in luminal specific ATAC-Seq peaks, many of the motifs identified have been well-studied, such as those from the GATA, FOX, and ETS families. Among them, NPAS has not been studied, and is therefore relatively novel.
3) Examination of the recent NPAS2 binding profile (HEPG2 cells) using the latest ENCODE data found that NPAS2 binds at the FOXA1 promoter region (shown below), but not at regulatory regions for basal marker genes. This suggested the possibility that NPAS2 may be an upstream regulator of FOXA1. Unfortunately, there is no commercially available antibody to study NPAS2 further.
4) Perhaps most intriguingly, we found that its expression level is significantly correlated with patient survival rates (current Fig. 5C), suggesting the translational relevance of NPAS2.

**New Figure 5A and 5B.** A) NPAS2 motif is enriched in both luminal-specific (P<0.0001) and shared ATAC-Seq peaks (P<0.001). B) NPAS2 binds at FOXA1 and GATA3 promoter in HepG2 cells.

*6/ When one looks at the function of TFs, we highly recommend analyzing them in the context of the cell line within the same subtype, i.e. knockout the TFs in luminal cells and see how they impact other TF binding to the expected sites via Chip-PCR and see if they compensate for each other? It is unclear why they overexpress a luminal TF NPAS2 in a basal model?*

We completely agree with the reviewer. As a matter of fact, we have tried both knock-out and knock-down of NPAS2 in luminal cells in the past several months. Unfortunately, the cells cannot survive the manipulation. We suspect that this is because NPAS2 is also an important circadian gene; therefore, its KD/KO might have other impact on the cell survival, which explains the lethal phenotype. As a last resort, we turned to the strategy of over-expressing this gene in basal cells.

*7/ And why would they use cell migration as an experimental endpoint (Figure 5H)? If they claim that overexpression of NPAS2 convert a basal into luminal phenotype, should they utilize a differentiation assay or at least look at the panel of basal/luminal markers (via qPCR)?*

We agree with the reviewer. In this revision, we over-expressed NPAS2 and performed qPCR in a panel of basal/luminal markers. Interestingly, we observed that it led to significantly decreased expression of four basal marker genes (showed below). However, we didn’t observe an increase of luminal marker gene expression. This suggests that the transition between the luminal and basal molecular subtype is complicated: overexpressing NPAS2 alone is not sufficient, and transitioning between different cell fates appears to be dependent on other key regulators.

**New Figure 5E**. RT-qPCR results for basal marker genes KRT5, KRT6A, STAT3, and TFAP2C for wildtype and NPAS2 overexpressed SCABER cell lines.

*8/ What’s the clinical implication by showing one luminal TF regulate expression of another luminal TF?*

The reviewer asks a question that is critical to the transcriptional regulation of urothelial biology. What are the major regulatory networks of epithelial differentiation? Why are these networks disrupted in cancer by carcinogens? The clinical implication of identifying NPAS2 as a potential regulator of primary luminal differentiation is that, once identified, this luminal TF can be targeted with drug therapy. In cases in which the cancers are less luminal and more basal, shifting the biology of the tumor to being more luminal by activating NPAS2 (and other required factors) could slow the growth of a basal cancer. Alternatively, 30% of muscle invasive tumors are luminal, with upregulated luminal pathways, and blocking the function of luminal tumors could improve survival of luminal cancers. For example, we now have a drug approved by the FDA targeting FGFR3 (erdafitinib), which is often mutated in luminal cancers. Yet, only 30% of FGFR3 altered tumors respond to this agent. Identification of NPAS2 as the potential regulator of luminal biology could facilitate development of small molecule targets to normalize the function of NPAS2 in luminal bladder cancer. This reasoning is now included in the revised discussion.

**Reference:**

1.	Kamoun A, de Reynies A, Allory Y, Sjodahl G, Robertson AG, Seiler R, Hoadley KA, Groeneveld CS, Al-Ahmadie H, Choi W, et al: **A Consensus Molecular Classification of Muscle-invasive Bladder Cancer.** *Eur Urol* 2020, **77:**420-433.

2.	Wang S, Lee S, Chu C, Jain D, Kerpedjiev P, Nelson GM, Walsh JM, Alver BH, Park PJ: **HiNT: a computational method for detecting copy number variations and translocations from Hi-C data.** *Genome Biol* 2020, **21:**73.

3.	Dixon JR, Xu J, Dileep V, Zhan Y, Song F, Le VT, Yardimci GG, Chakraborty A, Bann DV, Wang Y, et al: **Integrative detection and analysis of structural variation in cancer genomes.** *Nat Genet* 2018, **50:**1388-1398.

4.	Spielmann M, Lupianez DG, Mundlos S: **Structural variation in the 3D genome.** *Nat Rev Genet* 2018, **19:**453-467.

**Second round of review**

**Reviewer 1**

The revised manuscript has adequately addressed recommendations from the initial review of the paper. Publication is recommended.

**Reviewer 2**

1. With the small number of cell lines and samples, the authors will agree that it is essentially not possible to define "basal-specific" or "luminal-specific" 3D genome organization as they claim, i.e., referring to their own data showing such low concordance between their cell line and tumor samples. While we understand it is not cost effective to perform such profiling in large number of samples for a single lab, they are simply over claiming these so called "basal or luminal"-specific 3D genome organization, and will simply needed to be redefined when a larger sample size will be analyzed, which is not very useful?

2. A) NPAS2 is shared between luminal and basal from their raw data, now they claimed that because of the P value difference, it is primarily luminal specific? To me, both P values are highly significant which does not render NPAS2 more luminal specific (a bioinformatician needs to make such call).

B) Most importantly, the only functional experiment requested was not performed, and they simply argue that no antibody is available, and that knocking out NPAS2 in cancer cells rendering them not viable. NPAS2 deficient mice are viable and there are many tools, e.g., DOX-inducible knockdown or short term siRNA transduction is now available to drive home a simple point. Not only their point about overexpressing NPAS2 in a basal line is not physiological, if we extrapolate their point that overexpressing NAPAS2 turns a basal line into more luminal, then their #8 is very dangerous, will targeting NPAS2 turn a luminal cancer into basal (which is inherently more aggressive?). They simply did not perform the functional experiments requested and extrapolate.
